# A review and empirical findings of fasciae and muscle interactions in low back pain

**DOI:** 10.3389/fphys.2025.1604459

**Published:** 2025-09-10

**Authors:** Robbert N. van Amstel, Guido Weide, Eddo O. Wesselink, Karl Noten, Karl Jacobs, Annelies L. Pool-Goudzwaard, Richard T. Jaspers

**Affiliations:** ^1^ Laboratory for Myology, Department of Human Movement Sciences, Faculty of Behavioural and Movement Sciences, Amsterdam Movement Sciences, Vrije Universiteit Amsterdam, Amsterdam, Netherlands; ^2^ Department of Human Movement Sciences, Faculty of Behavioural and Movement Sciences, Neuromechanics, Amsterdam Movement Sciences, Vrije Universiteit Amsterdam, Amsterdam, Netherlands; ^3^ Fysio Science Department, Fysio Physics Group, IJsselstein, Netherlands; ^4^ Department of Medical Biology, Section Clinical Anatomy & Embryology, Amsterdam Reproduction & Development Research Institute, Amsterdam UMC, University of Amsterdam, Amsterdam, Netherlands; ^5^ Department of Oral Pain and Dysfunction, Functional Anatomy, Academic Centre for Dentistry Amsterdam (ACTA), University of Amsterdam and VU University Amsterdam, Amsterdam, Netherlands; ^6^ SOMT, University of Physiotherapy, Amersfoort, Netherlands

**Keywords:** physical therapy modalities, fascia, muscle, skeletal, connective tissue cells, biomechanics

## Abstract

**Background:**

Low Back Pain (LBP) is a global musculoskeletal disorder affecting quality of life, with 90% of cases categorized as nonspecific, indicating that the underlying cause is unknown. One of the current treatment modalities that physiotherapists use are fascia tissue manipulations (FTMs), such as soft tissue mobilization, myofascial release, and elastic tape, to enhance joint mobility and muscle flexibility in LBP individuals.

**Purpose:**

This review and experimental research explore the hypothetical mechanisms of FTMs using Skin Displacement (SKD), either by hand or with elastic tape.

**Methods:**

Several hypotheses regarding the working mechanisms of FTMs are discussed through inductive reasoning based on literature and new experimental results using ultrasonography and cadaver dissection. In this paper, stiffness is defined as the ratio of the applied force to the resulting strain, based on Hooke’s law. We focus on the role of lumbar fasciae and skeletal muscles, as well as the linkages between skin, fasciae, skeletal muscles, and joints, including the SKD-induced stress transmission between these structures. Furthermore, we discuss how the mechanical properties and stiffness of these structures can be altered.

**Results:**

The skin connects densely to the fasciae, back muscles, and spine, contributing to the stiffness of structures in the lumbar region. SKD maneuvers transmit stress to deeper tissues, causing strain and displacement of the thoracolumbar fascia, back muscles, and arthrofascia. These deformations may alter the active and passive mechanical properties of deeper tissues including fascia and muscle, by triggering stress-relaxation as well as structural adaptation.

**Conclusion:**

This paper provides indications that the skin is strongly connected to the thoracolumbar fascia, back muscles, and spine. These connections are possibly enhanced in patients with LBP. Stress applied to the skin by SKD maneuvers is shown to be transmitted to the underlying anatomical structures via these connections and can alter the stiffness of fasciae and skeletal muscles. The working mechanisms of FTMs potentially alter the quantity and composition of matrix components, as well as the contractile activity of muscle fibers, and traction forces of (myo)fibroblasts and other cells within the matrices. FTM-induced stress and alterations in anatomical structures not only improve joint mobility but also promote regeneration and tissue adaptation via various mechanisms resulting in pain relief.

## Introduction

Low Back Pain (LBP) is a common musculoskeletal disorder impacting quality of life (Mutubuki et al., 2020). It is the primary cause of Years Lived with Disability (YLDs) and Disability-Adjusted Life Years (DALYs) worldwide (Hurwitz et al., 2018; James et al., 2018). The incidence of LBP ranges from 0.02% to 7.0%, with lifetime prevalence reaching approximately 80%, and the prevalence ranges from 1.4% to 20.0%, higher in high-income countries (Fatoye et al., 2019; Chen et al., 2022; Ferreira et al., 2023). Ninety percent of LBP cases are nonspecific, with unknown causes (Bardin et al., 2017; Premkumar et al., 2018). Common symptoms include pain from the lowest rib to the gluteal area and upper leg (Kreiner et al., 2020). While psychosocial factors contribute to chronic LBP, biomedical aspects are also important (Cholewicki et al., 2019; Otero-Ketterer et al., 2022). The clinical presentation of chronic LBP is often linked to soft tissue changes, although some debate remains about the role of psychosocial factors in pain (Maher et al., 2017). One such soft tissue change that is debated involves the lumbodorsal fasciae and lumbar muscles (Wilke et al., 2017; Hodges and Danneels, 2019). Lumbar muscle contraction activity and spinal mobility differ in individuals with LBP compared to pain-free individuals (Moissenet et al., 2021). In healthy individuals, erector spinae muscles relax at maximal trunk flexion and extension, but in LBP patients, the activity of these muscles increases which is associated with increased thoracolumbar fascia stiffness and limited spinal mobility (Colloca and Hinrichs, 2005; Gouteron et al., 2021; Brandl et al., 2022; Brandl et al., 2023b).

Physiotherapists treat LBP by increasing joint mobility and muscle flexibility (Hernandez-Lazaro et al., 2022), often using hands-on techniques (Buchbinder et al., 2020). These techniques aim to restore muscle and fascia function and optimize joint mobility by addressing the fasciae and muscles to improve flexibility and movement (Brandl et al., 2021; Wu et al., 2021; Tamartash et al., 2022c). These techniques suggest to affect interconnected anatomical structures, including skin, fasciae, muscles, and neurovascular tracts (Noten and Van Amstel, 2024). Indeed, mathematical geometric modeling assumes that forces on the skin are transmitted to deeper anatomical structures (Chaudhry et al., 2007; Chaudhry et al., 2014). Novel hands-on techniques, such as Fascia Tissue Manipulations (FTMs), are thought to release fascia-muscle adhesions and reduce fascia and muscle stiffness by enhancing fascial compliance and mobility [25–28]. FTMs, including manual FTMs such as soft tissue mobilizations and the non-manual FTM of elastic taping, are hypothesized to change tissue stiffness, increase blood circulation, and restore fascia and muscle properties (Barnes, 1997; Simmonds et al., 2012). Soft tissue mobilizations are reported to soften the tissue (Tamartash et al., 2022b), while and elastic tape *in situ* has been postulated to continuously deform fascia and muscles (Pamuk and Yucesoy, 2015).

Although some evidence is conflicting, several systematic reviews suggest that FTMs may have positive effects on pain and disability in musculoskeletal disorders, including LBP (Ajimsha et al., 2015; Cheatham et al., 2015; Vanti et al., 2015; Laimi et al., 2018; van Amstel et al., 2021). While insights into FTMs’ mechanisms exist, scientific proof is indirect and based on theoretical models (Van Pelt et al., 2021; Ghorbanpour et al., 2023; Colonna and Casacci, 2024). A recent study showed that lumbodorsal skin displacement (SKD), as a derivative of FTM, significantly affects joint mobility instantaneously in healthy individuals (van Amstel et al., 2022). The effects on joint mobility varied by SKD location and direction (van Amstel et al., 2022). This suggests that physiotherapists using SKD-induced stress can manipulate joint mobility instantaneously, although data on SKD effects in LBP patients is lacking.

This review aims to provide an overview of the hypothetical mechanisms underlying SKD-induced stress in the treatment of LBP to optimize clinical parameters and discuss their potential involvement of FTMs in musculoskeletal disorders in general. To this end, we conducted a narrative literature review on the effects of various FTMs on lumbodorsal fasciae and muscles, supplemented by exploratory observational *in vivo* and *ex vivo* studies of anatomical structures and the shear strain caused by SKD-induced stress between the skin and underlying anatomical structures.

## Methods

### Hypotheses

This review examines three hypotheses on the potential mechanisms of FTMs, defined as interventions targeting fasciae, including manual FTMs such as soft tissue mobilizations, myofascial releases, and the SKD maneuver, as well as the non-manual FTM of elastic taping. FTMs acting manually on the skin are termed SKD-induced FTMs, while the elastic tape is simply referred to as such. These hypotheses are based on literature findings (Supplementary Material S1) and novel empirical, preliminary data from our research group. The review focuses on these hypotheses within the lumbar region, with particular emphasis on individuals suffering from LBP.

• Hypothesis 1: Prolonged inflammation may lead to the accumulation of adipose tissue and fibrosis, which can alter tissue thickness and increase tissue linkage density. This alteration potentially results in altered stiffness distribution within and around paraspinal muscles. Stiffer tissues reduce strain ability and limit fascial sliding mobility under spinal flexion and extension.
• Hypothesis 2: SKD-induced FTMs involve pressure as well as tensile and shear forces applied to the skin, which are transmitted to underlying anatomical structures, causing stress and strain in these anatomical structures.
• Hypothesis 3: The stress induced by SKD has the potential to alter the mechanical properties of anatomical structures underlying the skin. Moreover, SKD-induced stress may also alter the mechanical properties of fascia, thereby reducing excessive stress within and between fasciae, muscles, myofibroblasts, and specialized sensory cells. It is hypothesized that these alterations result in the relaxation of the fasciae and/or skeletal muscles, which is expected to increase the joint range of motion, reduce nociception, and thereby pain intensity. In addition, it is expected that the effectiveness of FTMs depends on both the location and direction of the SKD-induced FTM.

The validity of these hypotheses is discussed through a narrative review, for which we used specific search strings to identify studies in PubMed and Google Scholar published between 2000 and 2025, providing relevant information on the hypothetical mechanisms underlying FTMs, which are outlined in the Supplementary Material. The first 100 results were screened for relevance to the research questions. Additionally, references from identified papers were also reviewed for useful information (RVA and RTJ) (Haddaway et al., 2015). Each hypothesis section includes a summary of findings, highlighting evidence gaps and suggestions for future research.

**FIGURE 1:**
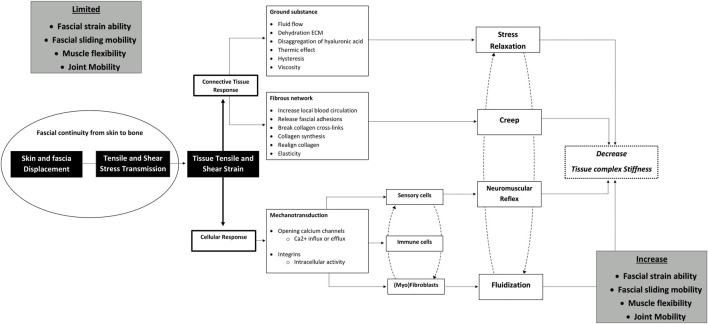
Schematic illustrating the potential mechanisms through which fascia tissue manipulations improve clinical outcomes in low back pain. Hypothesized working mechanisms of fascial tissue manipulations used in clinical practice to approach musculoskeletal disorders. The model is grounded in a skin displacement-induced fascia and muscle tissue principle in which moving the lumbar skin induces tensile, shear, and strain stresses which are transmitted to the underlying tissues. These stresses affect the fascia forming a connective tissue scaffold, leading to stress relaxation and creep in viscoelastic tissues. Subsequently, tensile and shear stresses induce cellular responses through mechanosensitive ion channels and transmembrane integrin signaling pathways. These signaling cascades result in a decrease in (myo)fibroblastic tensile contraction or muscle relaxation via neuromuscular reflex pathways. These effects together are presumed to result in a decrease in overall tissue stiffness, which will promote fascial straining, fascial sliding mobility, the direction and magnitude of myofascial force transmission, muscle activity, and joint mobility.

First, this review will provide background information on the anatomy, histology, physiology, and mechanics of the fasciae and muscles of the lower back, as well as key determinants that may be linked to limitations in lumbar mobility. This information supports the evaluation and discussion of the proposed hypotheses. Secondly, we explain the mechanical behavior of the lumbodorsal fasciae and how it mechanically connects the skin to the underlying anatomical structures. Then the hypotheses are discussed. Each hypothesis consists of two parts: a review part and a part with empirical observations. Finally, we evaluate the clinical implications, presenting an overview (Figure 1) of the evidence supporting or challenging each hypothesis.

## Determinants of lumbar tissue stiffness and joint movement and their alterations by cellular and tissue adaptation

### Terminology and definition

**FIGURE 2:**
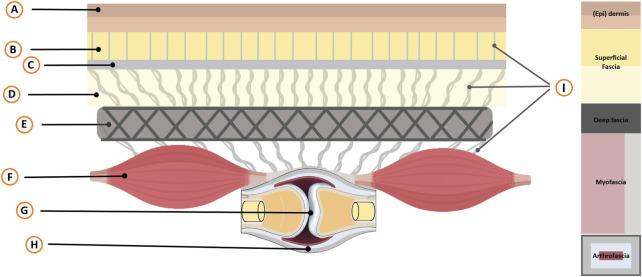
Schematic overview of the skin-arthro-myofascial complex. This schematic provides an overview of anatomical structures surrounding a joint from the skin to the bone that are mechanically interconnected. It encompasses the joint (Arthro) along with the muscles (Myo) and the fibrous connective tissues (Fascial; superficial-deep-myo-and arthrofascia) that are connected to the (epi)dermis. Legend: A, Epidermis and Dermis; B, Superficial adipose tissue; C, Superficial fascial membrane; D, Deep adipose tissue; E, Deep fascial membrane (Deep fascia); F, Myofascia (i.e., epimysium, perimysium, endomysium); G, Bony joints; H, Arthrofascia (i.e., ligaments, capsules, periosteum); I, Interfascial linkages (among others: retinacula cutis fibers and skin ligaments); Not displayed: Neurovascular tractus. F Depicts two fusiform muscles in series, illustrating anatomical and mechanical linkages and their role in intermuscular myofascial force transmission. The size of muscle F and joint G is intentionally reduced for illustrative purposes to help readers better comprehend the concept.

Fascia is a term that encompasses both an anatomical structure that could be classified into individual fascia, collectively known as fasciae, as well as a broader fascial system (Schleip et al., 2019b; Stecco et al., 2025). From skin to bone, fasciae encompass anatomical structures such as superficial fascia, deep fascia, myofascia (including skeletal muscle fibers), and arthrofascia (joint capsules and ligaments), which are continuously (uninterrupted) interconnected and collectively form a fascial system (Figure 2). At its core, fasciae are fibrous viscoelastic connective tissues that consist of matrices consisting of a ground substance being an amorphous gelatinous material formed by glycosaminoglycans, proteoglycans, glycoproteins, and hyaluronan, which are reinforced by fibrous materials such as collagen, reticular, and elastin fibers. Within these intricate matrices, cells such as myoblasts, fibroblasts, myofibroblasts, fibro-adipogenic progenitors, and fasciacytes reside, which play a crucial role in producing various components of the fascia (Chapman et al., 2017; Zullo et al., 2017; Stecco et al., 2018; Hinz et al., 2019; Schleip et al., 2019a). In addition, various specialized sensory cells are present, including mechanoreceptor cells, proprioceptors, and nociceptors, responding to alterations in chemical, thermal, and/or mechanical alterations (Stecco et al., 2007; Fede et al., 2022).

Fasciae consist of various dissectible fibrous connective tissues that attach, enclose, and separate muscle(s) (m, Mm), bones, and other internal organs (Schleip et al., 2019b; Stecco et al., 2025). Importantly, these individual fascial elements are interconnected, giving rise to a comprehensive and integrated fascial system (Adstrum et al., 2017).

### Fasciae form fascial continuity from skin to bone

The skin consists of three layers: epidermis, dermis, and hypodermis (subcutis) (Nash et al., 2004). The hypodermis contains superficial fascia, including a membrane, retinacula cutis fibers, and skin ligaments (Fede et al., 2025). This membrane separates deep and superficial adipose tissues, while retinacula cutis fibers and skin ligaments connect superficial fascia to the dermis and deep fascia (Al-Juhaishi et al., 2025). Observational studies have shown that the superficial fascia forms a fibrous tissue network (Herlin et al., 2015) of 10–20 mm thickness (Clarys et al., 2005), surrounded by a microvacuolar system with high viscoelastic properties (Guimberteau et al., 2010; Wong et al., 2016).

The deep fascia is a dense, fibrous layer with various phenotypes, including the general fascia, fascia lata, intermuscular septa, and thoracolumbar fascia (TLF). During prenatal growth, the TLF adapts to mechanical demands and forms three distinct layers (Abe et al., 2021): 1) ventral layer (anterior to m. quadratus lumborum), 2) middle layer (between m. quadratus lumborum and Mm. erector spinae), and 3) posterior layer (posterior to Mm. erector spinae). TLF separates superficial and deep back muscles (m. quadratus lumborum, Mm. erector spinae, and Mm. multifidi) via loose epimuscular connective tissue (Vleeming et al., 1995). The posterior layer is thickest in the sacral region and thinnest in the thoracic region (Marpalli et al., 2021). It comprises two distinct parts with perpendicular collagen fibers (Schuenke et al., 2012; Vleeming et al., 2012; Willard et al., 2012; Creze et al., 2018b). TLF collagen fibers merge with those of the epimysium in multiple trunk muscles (Schuenke et al., 2012; Willard et al., 2012; Vleeming et al., 2014).

Surface layers of skeletal muscles are formed by a three-dimensional (3D) connective tissue scaffold. This scaffold comprises the myofascia consisting of the epimysium, perimysium, endomysium, and tendon (Huijing, 2003; Schleip et al., 2012b). At some locations, the epimysium is thickened and forms aponeurotic connective tissues (e.g., erector spinae aponeurosis (ESA) reported to have different functions and mechanical properties than the epimysium (Fede et al., 2021). At the ends of the skeletal muscle belly, (i.e., at the myotendinous junctions), the aponeuroses turn into the tendon (Huijing, 2003). Histological studies have revealed that the endomysium, perimysium, epimysium, and tendons of skeletal muscles form a continuous (uninterrupted) interconnected honeycomb-like network of connective tissue (Huijing, 2008; Purslow, 2020; Sensini et al., 2021). Additionally, skeletal muscles attach, via epimuscular myofascial structures (Huijing, 2009), to the bones, periosteum, joint capsules, and ligaments (Maas and Sandercock, 2010; Willard et al., 2012; Creze et al., 2019).

The deepest fascia, known as the arthrofascia, links bones within joints and consists of joint capsules, ligaments, periosteum, and cartilage fibers (Noten and Van Amstel, 2020). Arthrofascial linkages are segmental (two bones) or regional (three or more bones) and influence passive joint motion (Widmer et al., 2020). Three arthrofascial connections exist: 1) synovial capsules (junctura synovialis) merging with periosteum, sometimes forming capsular ligaments (Martin et al., 2008); 2) ligaments (junctura fibrosa) linking bones functionally (Bejarano-Pineda et al., 2021); and 3) cartilage fiber linkages (junctura cartilaginea), e.g., annulus fibers in intervertebral discs (Schleip et al., 2012b). In the spine, these structures form functional spinal units (Bogduk, 2016), each consisting of vertebrae, intervertebral discs, ligaments, and other connective tissues. During spinal movements (flexion, extension, lateral bending, axial rotation), vertebral joints experience translation, rotation, and gliding (Cook et al., 2015). Arthrofascial structures provide passive joint stabilization and facilitate arthrokinematics (Butt et al., 2015).

In summary, fasciae form an interconnected continuous network from skin to bone. This facilitates the transmission of stress between anatomical structures and potentially allows the functional spinal unit to move as a cohesive whole.

### Microinjury and hypoxia lead to increased local stiffness and adhesions via inflammation

(Micro)injury to skeletal muscles and fasciae, including blood vessels, with the presence of a hematoma or seroma, increases local stialtered in individuals with LBP andffness of fasciae and/or skeletal muscles. This stiffness can be caused by hypercontraction of muscle fibers and myofibroblasts (Schleip et al., 2019a), as well as by increased local pressure due to fluid stagnation, leading to ischemia and hypoxia, which in turn may further enhance stiffness by promoting angiogenesis and altering local metabolic processes (Jiang F. et al., 2020). (Micro)injury and hypoxia trigger biochemical cascades resulting in the production of pro-inflammatory cytokines, increasing local extracellular ground substance and protein deposition essential for connective tissue regeneration. Suboptimal connective tissue recovery can lead to recurrent (micro)injuries, causing an individual to enter a vicious cycle of chronic prolonged inflammation, which may result in the accumulation of collagen cross-linkages, densification, adhesions, and increased tissue stiffness (Wilke et al., 2017; Zügel et al., 2018).

### Determinants of joint movement by fascia and skeletal muscle mechanical characteristics

Limitations in joint range of motion may result from the shortening or stiffening of anatomical structures, including 1) skin, 2) superficial fascia, 3) deep fascia, 4) skeletal muscles/myofascia, and 5) arthrofascia. These interconnected structures mechanically interact (Figure 2). Joint stiffness determinants include passive and/or active properties of tissues surrounding the joint. Passive stiffness is influenced by the fascia matrix components, intracellular cytoskeleton (e.g., muscle fiber titin) (Hwee and Jasper, 2016; Schleip et al., 2019a; Elosegui-Artola, 2021). Both structures exhibit viscoelastic behavior in response to mechanical loading with length- and history-dependent characteristics (Roszek and Huijing, 1997; Huijing, 2009). The active stiffness is determined by the actin-myosin interaction resulting in (con)tractile activity of the muscle fibers and cells within the fascia. The magnitude of the cellular contractile activity is the result of the number of filaments that are involved and the magnitude of the active state. Note that around a joint, it is the net effect of the stiffness of agonist and antagonist tissues that determines joint stiffness. In mechanics, stiffness (k) is defined as the ratio of the applied force (F) to the resulting change in length (ΔL), expressed as k = F/ΔL, in accordance with Hooke’s law.

Fasciae enclose fibroblasts, myoblasts, and myofibroblasts, which synthesize proteins for their extracellular matrix (Hinz et al., 2019). Collagen I and III dominate, with other types influencing mechanical properties (Kumka and Bonar, 2012). At rest, collagen fibers have a crimped pattern, which straightens under tensional stress, resulting in elastic deformation (Korhonen and Saarakkala, 2011). Collagen fibers absorb energy like a spring and recoil upon tension release (Schleip and Müller, 2013; Bell et al., 2022). Stress refers to applied force per unit area (σ = F/A). Under constant stress, fascia undergoes plastic deformation via stress-relaxation and creep (Purslow et al., 1998; Purslow, 2002). Collagen fibers regulate viscoelastic behavior through creep, stress-relaxation, and matrix reorientation (Purslow et al., 1998; Purslow, 2002). Dehydration induces rigid collagen crosslinks and hyaluronan tangling, altering viscoelastic properties (Matteini et al., 2009; Stecco et al., 2011; Jhorar and Lamba, 2022). Tissue viscosity, dependent on fluid content, impacts fascia compliance, recoil, deformability, sliding, tensile stress, and force transmission (Guimberteau et al., 2010; Stecco et al., 2011; Maas, 2019).

Concerning the passive muscular contribution to joint stiffness, the number of sarcomeres arranged in series and parallel within the muscle fibers critically determines passive muscle stiffness. The number of sarcomeres in series (i.e., including titin filaments) and myofascia stiffness determines both muscle fiber slack length and the slope of the passive stress-strain relation (Kruse et al., 2021), whereas the number of sarcomeres and the associated number of titin filaments arranged in parallel determine only the passive stiffness of the muscle fiber (Li et al., 2016). Both parameters are highly adaptive in response to mechanical stimuli and as such can be modulated substantially (Kruse et al., 2021). Regarding the active muscular contribution to joint stiffness, stiffness follows the parabolic relation of the sarcomere stress-strain relation determined by the magnitude of overlap between actin and myosin heavy chain filaments. The active contribution of fascia to joint stiffness occurs through cells within the extracellular matrix, such as myoblasts, fibroblasts, and myofibroblasts. These cells can contract in a smooth-muscle manner, thereby producing traction forces onto the extracellular matrix and as such tensioning specific parts within the fascia (Nosi et al., 2005; Schleip et al., 2006).

Traction by fibroblasts and myofibroblasts is coordinately regulated by biochemical factors such as Transforming Growth Factor Beta (TGF-β) (Schleip et al., 2019a) or by mechanical factors such as tensile or shear stress (Bakker et al., 2009; Krishnan et al., 2009). TGF-β is expressed by multiple cell types, such as monocytes, neutrophils, macrophages, myoblasts, and fibroblasts (Lawrence et al., 1984; Wynn and Vannella, 2016; Hillege et al., 2020). Histological research has revealed that TGF-β stimulates myofibroblastic and fibroblastic contractions exerting local tensions, which has been speculated to allow these contractile cells to mechanically load the extracellular matrix of the fascia via integrins (Schleip et al., 2019a; Schleip and Klingler, 2019).

Regarding mechanically induced cellular traction, it has been shown that cellular membrane deformation and mechanical stress applied to integrin elicit biochemical signals that cause cell traction (Ingber, 2003). Myofibroblasts and fibroblasts can contract in response to the activation of calcium (Ca^2+^) dependent calmodulin-myosin-light-chain kinase (Hinz et al., 2001; Tomasek et al., 2002; Levinson et al., 2004), which results in a rapid transient interaction between myosin and cytoskeletal F-actin or α-smooth muscle actin (Tomasek et al., 2002; Tomasek et al., 2006). The Rho kinase pathway also regulates cell traction. RhoA, a small Rho family protein, is activated by integrins and dystrophin-sarcoglycan complexes, triggering Rho kinase (ROKα/ROCK II) and inducing sustained (myo)fibroblastic contraction (Hinz et al., 2001; Tomasek et al., 2002; Levinson et al., 2004; Tomasek et al., 2006). In these ways, these cells strain and stiffen the fibrous materials within the fascia (Tomasek et al., 2002; Castella et al., 2010).

The stiffness and density of the linkages between fasciae, muscles, and bones affect the moment arm of fascia and muscle tissue crossing a joint, thereby influencing joint stiffness (Finni et al., 2023). The moment arm (r) is defined as the perpendicular distance from an axis of rotation to the line of action of a force and determines the mechanical leverage of a force (F) acting on a joint, with torque calculated as τ = r × F. The longer the perpendicular distance between the fascia or muscle line of action and the center of rotation of the joint, the higher the resistance to rotation. In the context of fascia and/or muscle stiffness (K), where force equals stiffness multiplied by resistance to length change (F = K × ΔL), the torque becomes τ = r × (K × ΔL), indicating that both stiffness, as resistance to length change, and the moment arm together influence joint mechanics (Macintosh et al., 1987). In this regard one should also consider the impact of myofascial mechanical interaction between structures, also referred to as myofascial force transmission (Huijing, 2009). Myofascial force transmission is the phenomenon that muscles can actively and passively transmit force not only via the muscle-tendon junction to the tendon but also via adherent epimuscular connective tissue linkages to adjacent and antagonist muscles, as well as to extramuscular connective tissues. Conversely, extramuscular connective tissues may affect each other laterally, including adjacent muscles. Via the myofascial connections, tensile and shear forces can be transmitted to other insertions at bony structures than those of the structures in which they were generated. In this way, the stiffness of the linkages between structures co-determines the net moment around a joint as the moment arm may increase or decrease depending on the directions in which forces are myofascially transmitted. Additionally, joint stiffness is affected by the relative position between adjacent structures and the direction of myofascial force transmission (Maas et al., 2003; Huijing, 2009; Yucesoy, 2010; Maas, 2019).

In summary, the intrinsic passive and active mechanical properties of fascia and skeletal muscle, along with their interaction, are important determinants of the optimal moment arms and maximal joint range of motion, as these factors will determine the tissue displacements necessary for free movement during daily activities. Understanding epimuscular force transmission and fascial mechanics is crucial for musculoskeletal function and the clinical application of FTMs.

### The role of mechanical loads on fascia and skeletal muscle adaptation in determining tissue stiffness around joints

When fasciae including cells experience external manual forces or contractile forces from muscle activity, stress arises with normal (tensile and compressive) and shear components. Tensile and compressive stress are interrelated, as pressure at one location can induce tension in surrounding collagen fibers and *vice versa*. Shear stress primarily occurs within fascia and muscles, as well as in the interconnecting layers at interfaces where anatomical structures shear relative to one another (Langevin, 2021). The resulting strain depends on the viscoelastic properties of the structure, such as stiffness and viscosity, which influence how much it deforms under the influence of a given force. Stiffer anatomical structures deform less than softer anatomical structures under the same stress. In addition, when the fatty connective tissue at anatomical interfaces becomes stiffer, it restricts the relative displacement of adjacent anatomical structures, such as the TLF and the ESA, with respect to each other thereby reducing shear strain at these interfaces (Pavan et al., 2014). At the cellular level, mechanosensitive ion channels and transmembrane integrins primarily respond to strain rather than to the absolute magnitude of force (Huijing and Jaspers, 2005). Therefore, in this paper we focus on both tensile and shear strains, as well as tensile and shear stress, since these are critical mechanical stimuli for mechanotransduction in fasciae and muscles, influencing cellular adaptation and extracellular matrix remodeling.

**FIGURE 3:**
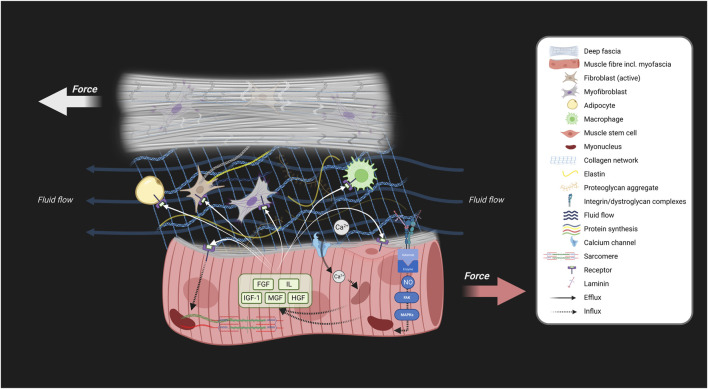
Schematic showing the involvement of myofascial interactions in skeletal muscle adaptation. Myofascial tensile and shear stress result from myofascial force transmission between structures, contributing to skeletal muscle adaptation. This force transmission can occur through tension in connective tissue linkages and fluid flow. This schematic explains how skeletal muscle growth and adaptation are influenced by mechanotransduction via extramuscular force transmission from fascia to intramuscular myofascia through the epimysium during joint movements. This process involves the conversion of these mechanical loads into biochemical signals through mechanosensitive ion channels (Ca^2+^), integrins, and dystroglycan-sarcoglycans, which activate or deactivate signaling pathways. As a result of the mechanical loading and biochemical signaling, various muscle growth factors and cytokines are synthesized and secreted by the muscle fibers, the myoblasts and other cells. Both tensile and shear forces activate mechanosensitive systems (depicted by black arrows). This activation stimulates biochemical signalling cascades for the synthesis of various growth factors and cytokines which are secreted into interstitial and epimuscular space. These signaling molecules may bind to membrane receptors of fibroblasts, macrophages myofibroblasts, adipocytes, and muscle cells, and contribute to stimulating cellular proliferation or alter the rate of protein synthesis resulting in cellular adaptation of skeletal muscle and fascia (white arrows). Note that impeded myofascial tensile and shear stress between fascia and muscle potentially negatively impacts skeletal muscle adaptation. Created with BioRender.com.

Skeletal muscles adapt their size to mechanical loads, in particular in response to resistance training and high strain (Huijing and Jaspers, 2005; Krzysztofik et al., 2019). During myofascial force transmission, epimuscular and intermuscular tensile and shear stress alter protein turnover in muscle fibers and other cells like fibroblasts and myoblasts, directly or through biochemical signaling, leading to muscle growth factors and cytokine secretion (Huijing and Jaspers, 2005). These factors may promote muscle adaptation in an autocrine and/or paracrine manner. Mechanical loads exerted on muscles, along with mechanical properties of surrounding fasciae, elicit signals within muscle fibers and surrounding cells which is referred to as mechanotransduction (Ingber, 2003). Cellular responses involve converting mechanical loads into biochemical signals through mechanosensitive ion channels, integrins, and dystroglycan-sarcoglycans. As a result, muscle growth factors and cytokines are synthesized and secreted (Figure 3). Tensile and shear forces elicit both similar and distinct physiological responses. In addition, a threshold of approximately 2–10 piconewton fluid tensile or shear stress is necessary to elicit a significant response, depending on the type of transmembrane molecule involved, such as integrins versus ion channels (Sun et al., 2016). For example, fluid shear stress stimulates nitric oxide production in osteocytes and C2C12 myotubes, while tensile stretch does not (Juffer et al., 2014). Additionally, fluid shear stress stimulates the influx of Ca^2+^, a process mediated by the glycocalyx, which activates mechanosensitive ion channels (Juffer et al., 2014). Myoblasts between the basal lamina and sarcolemma are subjected to tensile and shear loads during stretch-shortening (Haroon et al., 2021). Isolated myoblasts show upregulation of hepatocyte growth factor and interleukin-6 in response to fluid shear stress (Haroon et al., 2021). These findings suggest that shear stress is important in muscle adaptation and regeneration. However, changes in the quality and quantity of the fascia matrix cause densification (increase in collagen), and in more severe cases, fibrosis, which can lead to increased tissue thickness and stiffness (Pavan et al., 2014; Maas, 2019), reducing fascia/muscle mobility and fluid flow within the extracellular matrix (Langevin, 2021; Pratt, 2021). This may reduce mechanotransduction signaling, causing muscle atrophy, and shortening. Connective tissue also adapts to mechanical loads. TGF-β1, a key growth factor in connective tissue adaptation, modulates skeletal muscle and fascia. It is involved in the production of connective tissue components, important for muscle growth. During muscle fiber size changes, the fascia undergoes radial or longitudinal changes to maintain integrity with muscle fibers. Modulation of TGF-β1 receptor signaling optimizes myofascial adaptation. Chronic TGF-β1 expression may lead to increased collagen production in fibroblasts, myoblasts, and muscle cells (Pan et al., 2020; Shi et al., 2021; Hillege et al., 2022; Zhang et al., 2024). Knockdown of TGF-β receptors reduces fibroblastic growth factors and collagen (Hillege et al., 2020), enhancing myofiber regeneration (Hillege et al., 2022). This suggests that regulating TGF-β expression is critical for the balanced adaptation of muscle cells and connective tissue.

Taken together, tensile and shear strain between fasciae and myofibers play a crucial role in mechanotransduction. These forces activate signaling pathways that regulate muscle and fascia size while modulating passive stiffness. Increased fascial matrix densification, fibrosis, and stiffness may affect skeletal muscle morphology, particularly as myofascial force transmission induces the secretion of growth factors and cytokines (Figure 3).

### Interfascial sensorimotor complex monitors and coordinates agonist and antagonist motor unit activity

Fasciae are highly innervated tissues in the musculoskeletal system (Fede et al., 2021; Suarez-Rodriguez et al., 2022). Altered tissue stiffness increases susceptibility to microinjuries (Driscoll and Blyum, 2011), inflammation, nociceptor sensitization, impaired proprioception, and myofascial and muscle fiber denervation (Wilke et al., 2017; Hodges et al., 2021; James et al., 2022). These neurological impairments change the balance between agonist and antagonist muscle activity and passive muscle stiffness.

**FIGURE 4:**
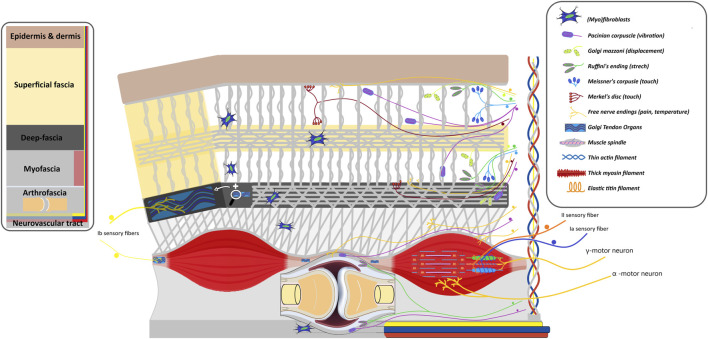
Schematic model illustrating the interactions between skeletal muscle, joints and fascia with implications for the sensorimotor system. This model presents key tissues of the musculoskeletal system and shows potential interactions between the (epi)dermis, superficial fascia, deep fascia, myofascia, skeletal muscles, arthrofascia, bones, mechanoreceptors, and motor neurons which are interfascially linked. This model provides a global impression of how sensory information may be detected within different tissues and how this information is translated into motor action. Impaired sensory functioning of these tissues can have an impact on adjacent tissues by eliciting sensory information that could disturb the motor response. Stiffening of superficial and deep fascia including the arthrofascia could tense and strain adjacent skeletal muscles, while strong muscle contraction can tense and strain these fasciae. Changes in relative positions of muscle fibers and fascia will cause tensions onto particular tissues in the musculoskeletal system which will be sensed by the mechanoreceptors eliciting a motor response (contraction and/or inhibition), causing (persistent) limitations in joint mobility.

The neurovascular tract penetrates the fasciae and muscles (Creze et al., 2018b). Mechanoreceptors, proprioceptors, and nociceptors are present in fasciae and muscles. Studies have shown a high density of mechanosensitive cells in the deep, superficial fascia, and arthrofascia, including Meissner’s bodies, Pacini bodies, Ruffini endings, Golgi-Mazzoni corpuscles, and free nerve endings (Yahia et al., 1992; McLain and Pickar, 1998; Stecco et al., 2007; Mense, 2019; Tomlinson et al., 2020; Laumonerie et al., 2021; Fede et al., 2022). Skeletal muscle proprioceptors, like muscle spindles and Golgi tendon organs, are near myofascial elements within muscles and adjacent to deep fascia (Fede et al., 2021; James et al., 2022). These sensory cells provide perceptions of movements and positions of skin, fascia, myofascia, muscles, and joints (Smilde et al., 2016) to regulate sensorimotor functions (Garofolini and Svanera, 2019) and body awareness (Schleip and Müller, 2013). Fasciae and muscles are optimal for sensory communication with the central nervous system, monitoring tension, and coordinating agonist-antagonist muscle activity (Fede et al., 2021). Sensory cells can be activated biochemically or mechanically through mechanosensitive channels. These channels, like T-type channels and Piezo2-gates, modulate neuronal excitability at low thresholds (Heppenstall and Lewin, 2006). They allow ions to pass through, affecting sensory cell activity (Bewick and Banks, 2021). This interaction forms an “interfascial sensorimotor complex” regulated by the central nervous system (Figure 4). Based on mechanographic data and cell density estimates, myofibroblasts in human and animal lumbar fascia and fascia lata generate contractile forces up to 445 mN/mm^2^ (Schleip et al., 2019a). Experimental evidence supports that myofibroblast contraction can mechanically trigger the opening of transmembrane channels in neighboring cells through mechanotransduction mediated by extracellular matrix tension and cell-cell junctions (Follonier et al., 2008). Hence, it is speculated that myofibroblasts embedded in fascia can generate contractile forces that either directly open mechanosensitive transmembrane channels or indirectly do so by stiffening the extracellular matrix through tensioning collagen fibers anchored to these channels in neighboring cells (Hinz et al., 2019). The mechanical stress resulting from myofibroblast contractions and subsequent deformation of adjacent cell membranes may lead to sensory cell activation, neuromuscular modulation, and likely influence joint mobility (Schleip et al., 2019a; Bewick and Banks, 2021).

Muscle spindles and Golgi tendons detect signals that influence skeletal muscle force. Possible mechanisms include the stretch reflex, which increases muscle tone in response to strain (Garofolini and Svanera, 2019), and skeletal muscle stress relaxation, which inhibits muscle activity due to sustained passive tissue tension (Garofolini and Svanera, 2019). In the lumbar region, the ESM relax as passive structures like the thoracolumbar fascia and spinal ligaments experience sustained tension, evident during forward bending (Floyd and Silver, 1955). This is the flexion relaxation phenomenon, where muscle contraction shifts to relaxation, and passive tissue tension supports trunk flexion (Zwambag and Brown, 2020). This suggests an interaction between passive and active components, with sensorimotor control coordinating agonist-antagonist muscle activity, which is ultimately influenced by passive tissue properties, determining the overall joint mechanics.

### Summary of determinants of lumbar tissue stiffness

Lumbar tissue stiffness and joint movement are influenced by several determinants. Fascia stiffness is regulated by fibroblasts and myofibroblasts, optimizing the fascia matrix. Fasciae coordinate motor unit activity through sensorimotor complexes that monitor fascial tension and muscle coordination. Myofascial tensile and shear stress drive muscle adaptation via mechanotransduction. Microinjury and hypoxia increase local stiffness by triggering muscle hypercontraction, myofibroblast activation, ischemia, and inflammation, altering the fascia and muscle matrix. The stiffness and density of the fascia matrix, along with its connections to muscles and bone, likely influence joint mechanics by affecting the moment arm of rotation and range of motion, which may in turn impact myofascial force transmission and motor unit function. Understanding these determinants is key to comprehending lumbar tissue stiffness and joint movement, underlying our hypotheses.

## Hypothesis 1: Prolonged inflammation may lead to the accumulation of adipose tissue and fibrosis, which can alter tissue thickness and increase tissue linkage density. This alteration potentially results in altered stiffness distribution within and around paraspinal muscles. Stiffer tissues reduce strain ability and limit fascial sliding mobility under spinal flexion and extension

In this section, the question is addressed whether fascial thickness, collagen content, and densification, as well as collagen cross-linking, are altered in individuals with LBP and whether these are related to increased TLF stiffness, muscle stiffness, reduced TLF sliding mobility, and muscle activity during joint motion. We discuss evidence from the literature, together with the results of our *ex vivo and in vivo* assessments, to explore whether there is a relation between lumbodorsal linkage density and relationships with fascial thickness supporting the above hypothesis.

### Prolonged inflammation leads to the accumulation of adipose tissue and fibrosis both within and outside paraspinal muscles

**FIGURE 5:**
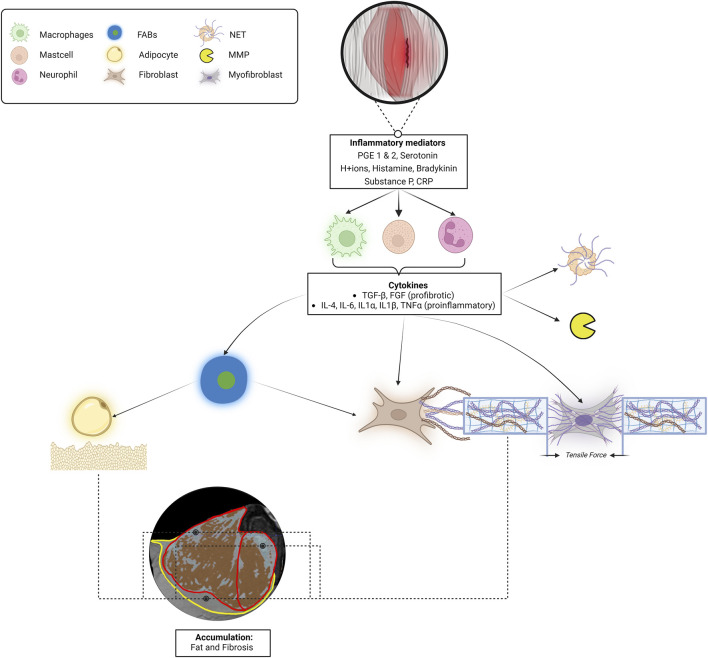
Schematic describing the process from local lumbar microinjury-induced inflammation to fat and fibrosis accumulation to increased tissue stiffness in the etiology of low back pain. When muscle and/or fascia tissues are injured, the encapsulated cells will secrete various biochemical mediators. These biochemical mediators trigger immune cells to release various cytokines, including TNFα and TGF-β. TGF-β stimulates myofibroblasts to contract, fibroblasts to synthesize collagen, and TNF-α attracts adipocytes, causing the accumulation of intramuscular and epimuscular fat and fibrosis. In the MRI image, the red boundaries represent the epimysium of the left ESM, and the yellow boundary corresponds to the left TLF. The red/brown areas indicate regions that depict muscle fibers, while the grey/blue areas suggest the possible presence of fat and fibrotic tissues. These observations are based on an analysis using a valid and reliable Gaussian mixture model applied to an MRI dataset from an asymptomatic individual recovered from low back pain (Wesselink et al., 2022; Wesselink E. et al., 2024). In addition, myofibroblastic contractions and the activation of neutrophil extracellular traps and matrix metalloproteinases contribute to altering connective tissue stiffness. The myofibroblastic tensile traction forces, in conjunction with this accumulation of fat and fibrosis, potentially increase the stiffness of the whole muscle-fascia complex. Abbreviations: PGE 1 and2, prostaglandins; CRP, C-reactive protein; TGF-β, Transforming Growth Factor-Beta; TNF-α, Tumor Necrosis Factor-Alpha; IL, Interleukins; NET, Neutrophil Extracellular Traps; MMP, matrix metalloproteinases; FABs, fibro-adipogenic progenitors; TLF, thoracolumbar fascia.

In case skeletal muscle and fascia are injured by (micro)injury, these tissues undergo a complex physiological recovery process in which multiple cells and physicochemical factors play a role (Huijing and Jaspers, 2005; Bentzinger et al., 2012) (Figure 5). During recovery from injury, the muscle stem cell enters a myogenic program, consisting of three main phases: inflammation (day 0–5), proliferation (day 5–21), and remodeling (day 21 <). Successful regeneration requires an orchestrated sequence of these phases (Bentzinger et al., 2013). In case this process is disturbed due to an overactivity of the innate immune system, this may lead to fibrosis and impaired muscle fiber regeneration.

During the inflammatory phase of muscle regeneration macrophages-1 and 2 (M1, M2), are activated by inflammatory mediators (prostaglandins, serotonin, H+ ions, histamine, bradykinin, substance P, and C-reactive protein) to infiltrate the interstitial space surrounding the myofiber (Ji et al., 2014). Immune cells like M1 produce pro-inflammatory cytokines, while M2 cells predominantly produce anti-inflammatory cytokines. Macrophages produce cytokines such as IL-4, IL-6, IL-1α, IL-1β, TGF-β, and TNF-α. These cytokines stimulate the formation of neutrophil extracellular traps and the expression of matrix metalloproteinases. In addition, they recruit or activate adipocytes, myoblasts, fibroblasts, myofibroblasts, and fibro-adipogenic progenitors (Bentzinger et al., 2012; Bentzinger et al., 2013; Zullo et al., 2017) (Figure 5). Fibro-adipogenic progenitors are specialized cells found in skeletal muscle tissue, activated in response to injury. They can differentiate into both adipocytes and fibroblasts, playing a crucial role in tissue repair and regeneration. Acting as the primary source of fat and fibrosis, fibro-adipogenic progenitors respond to pro-differentiation signals during muscle damage (Jin et al., 2019). The sequence of processes within the complex interactions between cells and physicochemical cues in the muscle stem cell niche is crucial for optimal tissue regeneration and adaptation, not only for that of the myofiber but also for the surrounding fasciae and proprioceptors (James et al., 2022). The prolonged presence of pro-inflammatory macrophages results in elevated levels of pro-inflammatory cytokines, including IL-1β, IL-6, TNF-α, and TGF-β in this specific area (Morris et al., 2020; Sanabria-Mazo et al., 2022). The sustained presence of the cytokines my cause a chronic accumulation of fat and fibrosis in this specific area (Chapman et al., 2017; Hodges and Danneels, 2019). This accumulation process may lead to variations in the distribution of fat and fibrosis, both within and outside the ESM, as illustrated in Figure 5. Consequently, this can result in increased local extracellular matrix stiffness, causing local strain distribution and recurrent microinjuries during muscle and fascia stretching.

The myofibroblastic tensile traction, in conjunction with this accumulation of fat and collagen, has the potential to make this lumbar musculoskeletal complex stiffer (Rahemi et al., 2015). If this stiffening occurs locally this may cause local strain distributions resulting in recurrent microinjuries during muscle and fascia stretch-shortening (Wilke et al., 2017; Zügel et al., 2018; Hodges and Danneels, 2019). If the musculoskeletal complex remains stiffened, these cells (e.g., myoblasts, fibroblasts, myofibroblasts, and fibro-adipogenic progenitors) may experience suboptimal loading, due to alterations in cellular mechanotransduction, resulting in reduced tensile stretch and shear stress. This can impede adequate muscle and fascia regeneration and hinder the development of optimal muscle compliance, as well as impair the viscoelastic behaviour of fascia. This, in turn, may impede normal muscle homeostasis (Hinz et al., 2001; Lacraz et al., 2015; van Santen et al., 2022).

### Increased lumbodorsal fascial thickness in individuals with chronic LBP

Understanding pathological changes in the lumbodorsal fascia can provide valuable insights into the underlying factors contributing to LBP.

A standardized ultrasonography method to assess the lumbodorsal fascial thickness, developed by Langevin et al. (2009), has been reported as a reliable technique for observing the lumbodorsal fascial thickness (De Coninck et al., 2018; Soares et al., 2021), and has been widely utilized in various studies testing the TLF thickness (Langevin et al., 2009; Larivière et al., 2020; Pirri et al., 2021; Tamartash et al., 2022a; Tamartash et al., 2022d). Using this method, variations in the thickness and morphology of the TLF among LBP and healthy individuals have been reported (De Coninck et al., 2018). A thickening of fasciae can potentially imply an enhanced stiffness of the lumbodorsal fascia, thereby affecting its mobility.

The change in TLF thickness after injury by a TLF incision has been investigated in porcine muscle. Ultrasonography revealed that the TLF on the injured side was thicker compared to that on the non-injured side (∼0.9 ± 0.2 mm vs. ∼0.7 ± 0.1 mm, p = 0.04) (Bishop et al., 2016). Individuals with chronic LBP who underwent scanning of the lumbodorsal fasciae by ultrasonography showed a substantially increased thickness of the TLF on both the left and right side (Langevin et al., 2009; Pirri et al., 2023). Furthermore, in healthy individuals, a substantial difference in TLF thickness was observed between the left and right sides; however, this discrepancy was not observed in individuals with LBP, indicating TLF densification and potentially a loss of TLF anisotropy (directional stiffness variations) (Pirri et al., 2023). Based on ultrasonography, the increased thickness of the TLF in LBP has been suggested to be the result of enthesopathy (inserting point inflammation) (Tabesh et al., 2021). These findings suggest that microinjuries and subsequent adipocyte infiltration may lead to the thickening of the TLF.

Ultrasonography studies in individuals with chronic LBP have reported an association between increased muscle fatty infiltrations in the lumbar multifidus muscle and elevated thickness of the TLF as well as epimuscular connective tissues surrounding the abdominal lateral wall muscles (Larivière et al., 2020; Larivière et al., 2023). These findings suggest a relation between the remodeling of lumbar fasciae and muscles and their potential association with increased lumbar stiffness in chronic LBP. However, to the best of our knowledge, a detailed composition of fasciae and muscles and alterations in their mechanical properties in LBP are unknown.

### Altered fascial sliding mobility during joint motion in individuals with LBP

TLF compliance and sliding mobility are argued to be pivotal for adequate trunk mobility in LBP individuals (Soares et al., 2021). However, a higher linkage density between lumbar anatomical structures is expected to result in smaller TLF compliance and displacements, as well as reduced shear strains between the TLF and ESM, due to the increased stiffness of the linkages between these structures. As a consequence, force transmission between the TLF and ESM increases, while shear strain decreases, thereby limiting joint mobility.

**FIGURE 6:**
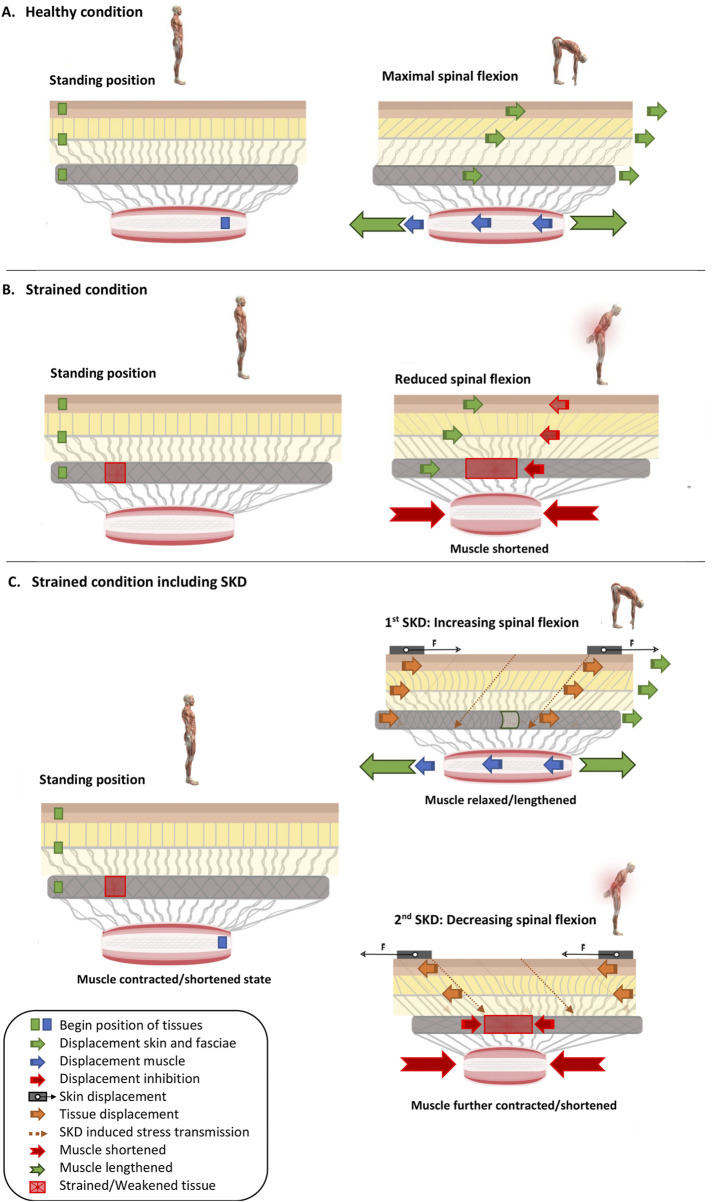
Skin Displacement by fascia tissue manipulation influences muscle and fascia mechanics in situations with and without low back pain. Several conditions are represented: **(A)** without LBP, **(B)** with LBP, and additionally either without **(A,B)** or with **(C)** ongoing SKD. Different symbols are present in the images as mentioned in the legend. In images **(A–C)**, the green squares (Dermis, TLF, and SF) and blue squares (Muscle) with arrows indicate the displacement direction of these anatomical structures. Red arrows show the counter-displacement of the anatomical structures caused by muscle contraction to protect the red square representing the injured tissue. The thick orange arrows in image **(C)** represent the transmission of force (indicated by the thin dashed orange arrow) due to skin displacement. **(A)** Healthy condition in which a subject moves from a standing position to maximal trunk flexion while the superficial fascia, thoracolumbar fascia slide over the erector spinae muscle. **(B)** Strained condition (injured tissue, caused by excessive stress such as overstretching and/or overuse, resulting in a reduction of the tissue’s resistance to strain) in which a subject moves from standing position to maximal trunk flexion, while the superficial fascia, thoracolumbar fascia slide over the erector spinae muscle. However, noxious stress is registered, and back muscles contract to protect against further loading of the strained and sensitized thoracolumbar fascia. **(C)** Strained condition in which during the movement from a standing position to maximal trunk flexion, skin displacement as a Fascia Tissue Manipulation is performed. From standing position to maximal trunk flexion, the skin is displaced in such a way that the strained and sensitized tissue is unloaded, leading to muscle relaxation and lengthening, thereby increasing the spinal range of motion. However, if the strained and sensitized tissue is overloaded, the skin displacement leads to continuation and more intensified muscle contraction and shortening, thereby reducing the spinal range of motion. Note that this is a fictitious example, and the direction of the skin displacement is undefined (i.e., the skin displacement could be in the left or right direction, whether or not in combination with cranial or caudal displacement).

In a healthy condition, when flexing the spine, the superficial fascia and TLF are expected to be strained and will be subjected to shear stress causing resistance towards elongation. Shear strain is defined as the relative horizontal displacement (x-axis) over time, calculated as the difference in position (P) between two anatomical structures, specifically the TLF (P_TLF) and the ESA (P_ESA), divided by the vertical distance between them (Δ_TLF–ESA). This ratio indicates the extent to which the anatomical structures shear past each other per unit of vertical separation and serves as a measure of mobility. Mathematically, this is expressed as a percentage of shear strain: Shear strain (%) = (|P_TLF − P_ESA| × 100)/Δ_TLF–ESA (Langevin et al., 2011). According to the findings of Langevin et al. (2011), in a healthy condition, during spinal flexion, the TLF extends and slides over the deep back muscles with a shear strain of approximately 70.2% ± 3.6%, facilitating normal fascial mobility (Figure 6A). In a strained condition, which can occur in all anatomical structures, it is hypothesized that muscles will contract to prevent the strained tissue from further loading (Figure 6B).

During passive lumbar flexion in LBP individuals, the shear strain between TLF and ESA determined by ultrasonography has been shown to be substantially reduced (15.7%–20%) compared to that in healthy controls (Langevin et al., 2011; Tomita et al., 2025). The shear strain magnitude of the TLF over the ESA is correlated with the epimuscular connective tissue thickness (r = −0.45), its echogenicity (r = 0.28), trunk flexion range of motion (r = 0.36), and extension range of motion (r = 0.41) (Langevin et al., 2011). In addition, significant differences in TLF shear strain have been measured during the transition from trunk flexion to the neutral straight position (Brandl et al., 2023b). This trunk motion was observed in both trunk extension tasks and deadlifting exercises among track and field athletes, individuals with LBP, and healthy controls. Track and field athletes exhibited the largest TLF shear strain (−37.6%), followed by untrained healthy individuals (−26.4%), while individuals with LBP had minimal TLF shear strain (−2.7%) during trunk extension tasks and deadlifting exercises (Brandl et al., 2023b).

In conclusion, the observed increase in TLF thickness and reduced shear strain in individuals with LBP, compared to matching controls, suggests that the increase in TLF thickness in these patients may result in increased intra-TLF collagen thickness, density, and stacking (Hedley, 2022). This could enhance TLF stiffness, thereby reducing TLF strainability, increasing force transmission between superficial back muscles, and reducing TLF sliding mobility over the ESM during trunk motions.

### Testing hypothesis 1: dense lumbodorsal linkages observed from the skin to the spine in a cadaver and increased *in vivo* fascial thickness in two individuals

To investigate potential chronic LBP-related changes and adaptations of lumbodorsal connective tissues and the linkages between them, we assessed the different fasciae in the lower back regions *in vivo* and in a cadaver specimen (*ex vivo*) by using three different observation techniques: 1) segmentation of 3D images of an anatomical model (Visible Human Male (NIH, 2013)), 2) visualization of lumbodorsal fasciae and muscles in a fresh-frozen cadaver, and 3) 2D ultrasonography analysis. The 2D ultrasonography images were obtained from a non-embalmed male freshly frozen cadaver and from 2 male subjects, one with (age 39, 178, 78 kg, BMI 24.5) and one without LBP (age 22, 189, 83 kg, BMI 23.2) using a 12–2 MHz linear array transducer (Arietta Prologue; Hitachi Ltd., Tokyo, Japan). The fresh-frozen cadaver was stored at −20°C and was thawed at room temperature 24 h before the investigation. This study was approved by the Scientific and Ethical Review Board (METC) of the VU University Medical Center Amsterdam, Vrije Universiteit Amsterdam (CTB- 2017.098 (A2017.457) (Supplementary Material S2).

**FIGURE 7:**
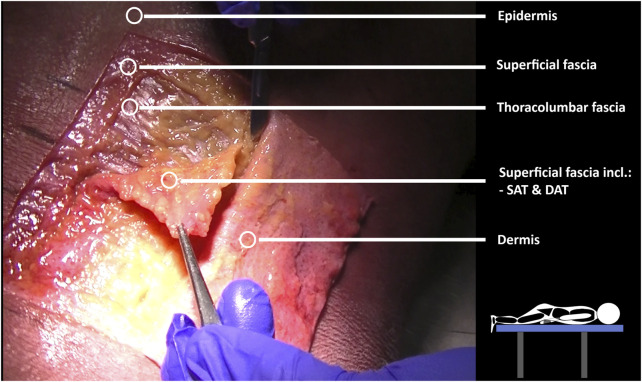
Mecchanical interactions between fascia and skeletal muscle by layer-by-layer cadaver fascia dissection. Dissections were performed on a male cadaver. The cadaver was positioned prone on a table to investigate the fascial subcutaneous linkages of the lumbodorsal tissues, extending from the skin to the spine. Subsequently, each layer (epidermis/dermis, superficial fascia, thoracolumbar fascia, erector spinae aponeurosis) was dissected to form a rectangular section at the T12–L3 level.

**FIGURE 8:**
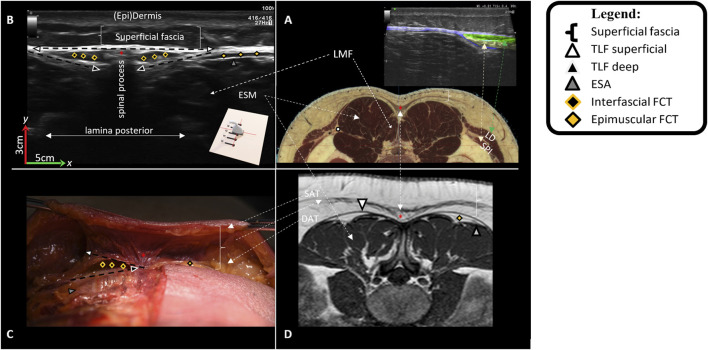
Lumbodorsal fascia anatomical tissues from a transversal view. In this figure, Image **(A)** represents a transverse MRI image from a Visible Human Male, showing the lumbar tissues at the height of L2-3. Image **(B)** represents a transverse ultrasonographic image of a healthy individual at the height of L2-3. Image **(C)** represents a transverse image of a freshly frozen human male cadaver dissected at the height of L2-3. Image **(D)** is an MRI image (T1, TR 707, TE 11) at the height of L2-3 of a nonspecific low back pain female patient. Image **(A)** displays the following lumbar tissues: (epi)dermis, superficial fascia, SAT (superior adiposity tissue), DAT (deep adiposity tissue), LD (latissimus dorsi muscle), SPI (serratus posterior inferior muscle), LMF (lumbar multifidus), ESM (erector spinae muscle), *CIFT (central interfascial triangle), and lateral raphe within the TLF. Certain tissues, while not clearly visible in Image **(A)**, become visible in Images **(B–D)**. These include the TLF (thoracolumbar fascia, consisting of superficial and deep lamina), interfascial fat lying between the superficial and deep lamina of the TLF, and epimuscular fat lying between the TLF and the erector spinae aponeurosis (ESA).

**FIGURE 9:**
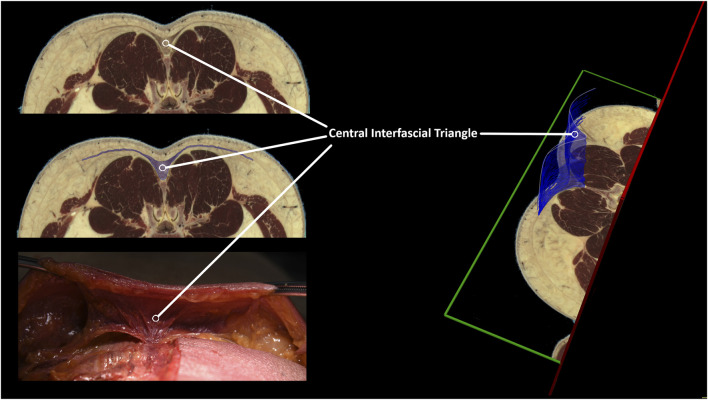
Identification of a central interfascial fascia triangle between the skin, erector spinae muscle, and lumbar spine. The lumbar tissues in the lumbar spine were examined using an MRI anatomical model (Visible Human Male) (NIH, 2013). Regions of interest were defined, encompassing the lumbar central interfascial triangle, which was manually segmented with transverse anatomical cross-references from the upper endplate of L2 to the lower endplate of L3. The 3D representation of the central interfascial triangle is the result of our segmentation, spanning from L3 to L2. Although not shown in this figure, this triangular-shaped tissue is present from S1 up to T12. The segmentation was performed using MITK v2022.10.

The dissection and 3D model analysis revealed a strong connection between the skin, superficial fascia, and the superficial lamina of the posterior layer of the TLF (Figure 7), as well as the spinous processes and supraspinal ligaments (Figure 8). The lumbodorsal central interfascial triangle (CIFT) was identified at the lumbar vertebrae (T12-L5), where the superficial fascia forms a dense collagenous structure (Figure 9), not to be mistaken for the lateral raphe (Schuenke et al., 2012). Additionally, interfascial fat was observed between the superficial and deep layers of the TLF, and between the deep lamina of the TLF and the ESA (Figures 8B–D). Ultrasonography measurements indicated a thicker TLF in individuals with LBP, suggesting thickening of the TLF laminae and associated fat. The mean cross-sectional area (CSA) of the TLF in the LBP case was 122 mm^2^, compared to 90 mm^2^ in the healthy case, while the CSA of the hypodermis including the superficial fascia was 243 mm^2^ in the LBP case, compared to 258 mm^2^ in the healthy case. These findings provide valuable insights into the dense lumbodorsal linkages between the skin, TLF, back muscles, and lumbar spine in these cases. The observed changes in the TLF in the LBP individual warrant further investigation into the role of connective tissue adaptations in chronic LBP.

### Conclusion and perspective for future research on fasciae thickness and altered linkages in individuals with LBP

Several studies have shown that lumbar anatomical structures are interconnected, with variations in TLF thickness and connective tissue density. Both *in vivo* and *ex vivo* observations show strong linkages between the lumbodorsal skin, TLF, muscles, and lumbar spine, confirming continuity from skin to bone in humans. A dense connection from the skin to TLF, muscles, and spine via CIFT has not been reported before. These linkages likely influence mechanical interactions between tissues, warranting further exploration of collagen content and how changes contribute to lumbar stiffness.

Ultrasonography shows thicker TLF in individuals with LBP compared to healthy controls, possibly due to variations in fat and connective tissue. Since TLF cannot be distinguished from epimuscular fat in ultrasonography, thickness might be overestimated. Future studies should explore the association between epimuscular fat and LBP factors using advanced imaging. Investigating the composition of lumbodorsal interfascial and epimuscular connective tissues, particularly fat-to-collagen ratios, in healthy individuals and those with LBP is crucial for understanding fascia-related LBP and developing targeted therapeutic interventions. Increased thickness in these tissues may alter stiffness, impacting spinal mobility, making them a potential diagnostic target for treatment.

## Hypothesis 2: SKD-induced FTMs involve tensile and shear forces applied to the skin, which are transmitted to underlying anatomical structures, causing stress and strain in these anatomical structures

In general, the primary objective of FTMs is to apply stress to the anatomical structures underneath the skin, aiming to induce strain on these structures, thereby reducing their stiffness. During FTMs, the skin is subjected to shear forces, tensile stress, and/or pressure, subjecting the underlying connective tissues to normal, tensile, shear, torsion, and bending stress. Mathematical geometric modeling has shown that forces applied to the skin can strain and displace the fasciae, potentially altering the relative position of the TLF and back muscles, as well as the mechanical properties of the underlying fasciae (Chaudhry et al., 2008; Chaudhry et al., 2014). This has led to the hypothesis that the pressure as well as tensile and shear forces generated during FTMs are transmitted via the skin to the lumbodorsal superficial fascia, TLF, interfascial and epimuscular fat, back muscles, spinal arthrofascia, and the CIFT. These forces are expected to tense and strain the mentioned structures, causing the TLF to shear (and slide) over the ESM while influencing the relative positions between fasciae, skeletal muscles, and bones, with respect to each other. SKD-induced strain is expected to be direction-dependent, as cadaver studies have shown that the superficial fascia is stiffer in the mediolateral direction than in the cranio-caudal direction for both the abdominal and thoracic regions (Berardo et al., 2024). However, little is known about the impact of an SKD maneuver on the anatomical structures below the skin in both individuals with and without LBP.

### Testing hypothesis 2: Shear strain analysis by ultrasonography reveals SKD-induced transmission of stress to underlying structures, providing proof of the SKD principle

To demonstrate that SKD-induced stress transmits force from the skin to underlying structures, SKD maneuvers were performed on two individuals and one cadaver at location L3 (Supplementary Material S2). Four SKD maneuvers were performed by a physiotherapist with 11 years of experience. The maneuvers involved mediolateral SKD (right or left) and vertical SKD (cranial or caudal) above the right ESM in both neutral and flexed positions. The intensity of the maneuver was adjusted to the skin slack (i.e., the resistance of skin to displacement).

**FIGURE 10:**
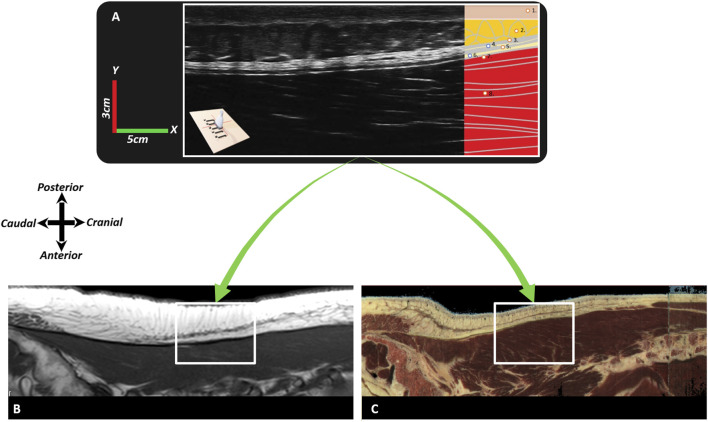
Typical images of the different fasciae in the lumbodorsal region of a healthy subject, a cadaver, and a patient with low back pain using ultrasonography or MRI. In this figure, Image **(A)** represents a sagittal ultrasonographic image (3–5 cm, y-x) *in vivo* of a healthy male subject. The ultrasonography probe was placed longitudinally above the right erector spinae, 2 cm laterally from the L2-3 spinal process. From superficial to deep, the following tissues are identified: 1) (epi)dermis, 2) superficial fascia/hypodermis, 3) superficial lamina of the TLF, 4) interfascial fat, 5) deep lamina of the TLF, 6) epimuscular fat, 7) erector spinae aponeurosis, and 8) erector spinae muscle. Image **(B)** represents an MRI (T1, TR 707, TE 1) of a nonspecific chronic LBP female individual, and Image **(C)** is an MRI model (Visible Human Male). The white square box represents the equivalent region (3–5 cm, y-x) as in Image **(A)**. Note that in Image **(A)** (ultrasonography), the superficial fascia is not as visible as in Images **(B,C)**; however, the thoracolumbar fascia and erector spinae aponeurosis are more visible in Image **(A)** and less visible in Images **(B,C)**.

Effects on the underlying structures were assessed using ultrasonography of the right side, recording images above the latissimus dorsi (LD) and ESM in sagittal (Figure 10) and transverse planes, with the probe placements as described in the literature (Langevin et al., 2011; Wong et al., 2017). Anatomical structures of interest include the lumbodorsal dermis, superficial fascia, TLF, LD, SPI, ESA, and ESM. Speckles were tracked using Kinovea, and tissue displacement was quantified using MATLAB (version 2023b, The MathWorks, Inc., Natick, MA, United States) (Van Amstel et al., 2025). Shear strain ratios were calculated between the dermis and each underlying anatomical structure.

**FIGURE 11:**
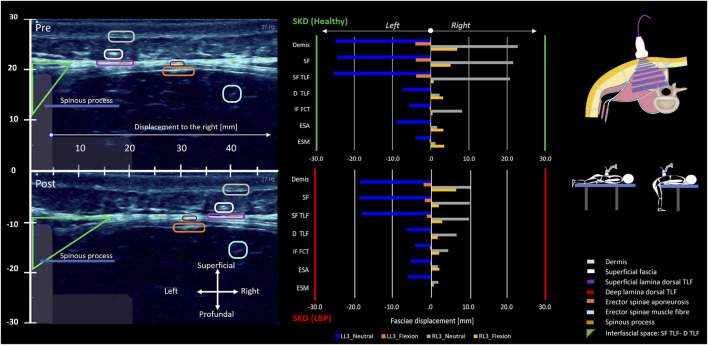
Lumbodorsal SKD-induced stress transmission to underlying tissues and their displacements and deformations. The two individuals, healthy (green y-axis) and LBP (red y-axis), were placed prone on a physio plinth. A foam block under the SIAS obtained 10° lumbosacral flexion, the neutral position (blue and grey). For the flexed position, subjects were positioned at the end of the physio plinth with legs on the floor and forearms on the plinth to maintain 60° spinal flexion (orange and yellow). Ultrasonography videos were recorded in both neutral and flexed positions. Post-analysis of the ultrasonography recordings was performed using speckle tracking analysis. With the obtained data, the absolute displacement in millimeters (mm) of each anatomical structure (epidermis, superficial fascia, TLF, ESA, ESM, SPI, LD) was calculated. Additionally, the shear strain ratio between the (epi)dermis and each anatomical structure was calculated. In the ultrasonography images, the region of interest for each anatomical structure is circled and corresponds to the colors in the legend. The images represent the absolute displacements of anatomical landmarks in mm due to the SKD maneuver to the right and left at location L3. The region of interest circles allow for visual estimation of the absolute displacement. The plots show the displacement results in mm in neutral (blue and grey) and flexed positions (orange and yellow). The absolute displacement of the anatomical structures is greatest superficially and decreases in depth. Shear strain ratios between the (epi)dermis, superficial fascia, and the TLF were small, indicating equal displacement. In contrast, higher ratios between the (epi)dermis and back muscles suggested a greater shear strain ratio and potentially higher shear stress (see Tables 1, 2). Abbreviations: SKD, skin displacement; SF, superficial fascia; D TLF, deep thoracolumbar fascia; IF FCT, interfascial fat; SF TLF, superficial thoracolumbar fascia; ESA, erector spinae aponeurosis; ESM, erector spinae muscle.

The results of the tests show that SKD-induced stress caused displacement of anatomical structures. The shear strain ratios were small between the (epi)dermis, superficial fascia, and posterior TLF, indicating equal displacement. Higher shear strain ratios were observed between the (epi)dermis and deep back muscles such as the ESA, indicating that superficial structures like the TLF shear/slide over the ESA. The displacement followed a hierarchical pattern, with the greatest displacement occurring superficially and decreasing in depth. The displacement was greater in the healthy individual than in the LBP individual and cadaver. Cadaveric findings showed that SKD-induced stress caused the superficial fascia and TLF to slide over the ESA due to the mobile epimuscular fat, while tension increased on the arthrofascia via the CIFT, confirming force transmission from skin to bone (Figures 7, 8, 11; Tables 1, 2).

**TABLE 1:** SKD principles: Mediolateral Skin-Myofascial displacement and shear-strain at L3.

Right SKD												Left SKD							
Layers	Plane L3	Depth		Neutral				Flexion				Neutral				Flexion			
		Healthy	LBP	Healthy		LBP		Healthy		LBP		Healthy		LBP		Healthy		LBP	
		mm	mm	ABS/mm	SSR	ABS/mm	SSR	ABS/mm	SSR	ABS/mm	SSR	ABS/mm	SSR	ABS/mm	SSR	ABS/mm	SSR	ABS/mm	SR
(Epi) Demis	T	*	*	22.8	*	10.0	*	6.9	*	6.3	*	−24.9	*	−18.2	*	−24.9	*	−1.9	-
Superficial fascia	T	6.7	4.9	21.6	17.6	9.8	4.7	5.2	25.7	1.9	90.8	−24.4	6.9	−18.4	−4.1	−24.4	1.3	−1.6	6.9
Superficial lamina TLF	T	8.6	7.3	20.7	23.4	9.6	5.6	*	*	*	*	−25.3	−4.7	*	*	−25.3	1.9	*	*
Profundal lamina TLF	T	8.7	7.4	8.2	122.2	6.4	48.8	3.2	42.4	1.6	49.3	−7.2	−197.5	−6.1	−162.6	−7.2	45.6	−0.1	−24.1
Interfascial triangle	T	8.6	8.1	8.2	169.3	4.3	70.5	0.5	75.0	2.0	63.5	−5.5	−219.7	−4.1	−173.0	−5.5	−43.3	−0.3	−19.8
Erector spinae aponeurosis	T	9.9	8.8	1.6	213.2	1.6	95.6	3.3	36.6	2.0	53.6	−8.8	−158.1	−5.3	−145.8	−8.8	−38.8	−0.2	−19.8
Mm. erector spinae	T	16.5	13.5	1.3	130.1	1.7	61.2	3.5	20.4	0.6	49.0	−3.9	−83.3	−5.9	−19.4	−3.9	−22.1	0.0	−13.9
Latissimus-TLF junction	CR	9.4	9.6	8.2	155.2	0.2	102.7	0.1	72.7	0.5	60.7	−8.9	−169.9	−5.9	−128.0	−0.9	−32.2	−0.6	−13.3
Latissimus dorsi muscle	T	8.9	8.7	3.4	217.6	1.0	103.7	4.3	29.7	0.4	68.3	−6.1	−210.7	−0.9	−200.3	−1.8	−24.4	−0.2	−20.3
Serratus post. Inf. muscle	T	9	10.4	3.4	215.2	1.5	82.3	4.3	29.3	1.9	233.7	−7.7	−190.9	−0.5	−164.3	−1.8	−24.1	−0.2	−17.0

This table represents the force transmission from the skin to the underlying anatomical structures on the right side (above the erector spinae muscles). It shows the tissue depth, which is the distance between the epidermis and the ROI-box of the anatomical structure. Additionally, it presents the absolute and shear strain ratios. The shear strain ratio is calculated by ∣P1−P2∣×100/D, where P1 is the epidermis and P2, P3, Pi, etc., are the underlying anatomical structures and D is the Depth of the tissue. The absolute and shear strain ratios are shown for both the right and left SKD maneuver in a neutral prone lying position and a standing flexion position. The lower the shear strain ratio, the smaller the shear deformation and possibly the shear stress experience by the specific anatomical structure due to SKD maneuver. It was not possible to differentiate between the superficial and profundal lamina of the TLF in trunk flexion.

T, transverse plane, CR, cross-rotation plan; mm, millimeters; Mm, Musculi; ABS/mm, absolute displacement in mm; SSR, shear strain ratio; LBP, low back pain individual; SKD, skin displacement; RL3, SKD mediolateral to the right at L3; LL3, SKD mediolateral to the left at L3; *, no data.

**TABLE 2:** Caudo-cranial Skin-MyoFascial displacement and shear-strain at right erector spinae muscle.

Cranial SKD												Caudal SKD							
Layers	Plane L3	Depth		Neutral				Flexion				Neutral				Flexion			
		Healthy	LBP	Healthy		LBP		Healthy		LBP		Healthy		LBP		Healthy		LBP	
				ABS/mm	SSR	ABS/mm	SSR	ABS/mm	SSR	ABS/mm	RS	ABS/mm	SSR	ABS/mm	SSR	ABS/mm	SSR	ABS/mm	SSR
(Epi) Demis	S	*	*	12.8	*	3.0	*	2.5	*	8.9	*	−2.4	*	−0.8	*	−0.9	*	−0.5	*
Superficial fascia	S	6.5	5.4	11.9	13.5	2.9	0.4	2.2	4.2	7.3	30.4	−2.4	−0.9	−0.7	−1.7	−0.8	−0.8	−0.5	−0.6
Superficial lamina TLF	S	8.9	8.1	9.3	38.8	2.6	4.6	2.1	4.1	6.8	25.9	−2.2	−2.5	0.1	−10.5	−0.9	−0.4	−0.5	−0.4
Profundal lamina TLF	S	9.1	8.3	0.3	137.6	1.6	16.7	*	*	*	*	−0.2	−24.5	−0.5	−2.7	*	*	*	*
Erector spinea aponeurosis	S	10.9	10.0	−3.3	147.4	1.0	19.7	0.4	19.0	0.0	78.2	−0.1	−21.5	0.0	−7.2	−0.2	−6.0	0.0	−5.3
Erector spinea muscles	S	14.7	15.9	−3.0	107.1	0.1	17.9	0.3	15.0	1.1	54.2	−0.1	−15.9	−0.2	−3.5	−0.3	−4.0	0.0	−5.0

This table represents the force transmission from the skin to the underlying anatomical structures on the right side (above the erector spinae muscles). It shows the tissue depth, which is the distance between the epidermis and the ROI-box of the anatomical structure. Additionally, it presents the absolute and shear strain ratios. The shear strain ratio is calculated by ∣P1−P2∣×100/D, where P1 is the epidermis and P2, P3, Pi, etc., are the underlying anatomical structures and D is the Depth of the tissue. The absolute and shear strain ratios are shown for both the right and left SKD maneuver in a neutral prone lying position and a standing flexion position. The lower the shear strain ratio, the smaller the shear deformation and possibly the shear stress experience by the specific anatomical structure due to SKD maneuver. It was not possible to differentiate between the superficial and profundal lamina of the TLF in trunk flexion.

S, sagittal plane; mm, millimeters; Mm, Musculi; ABS/mm, absolute displacement in mm; SSR, shear strain ratio; LBP, low back pain individual; SKD, skin displacement; Cr_ESM, cranial SKD over right erector spinae muscle; Ca_ESM, craudal SKD, over right erector spinae muscle; *, no data.

### Conclusion and perspective for future research on SKD-induced FTMs involving tensile and shear forces on anatomical structures

Although the abovementioned ultrasonography data are based on a small sample of subjects, the observations indicate that 1) the TLF is thicker in the LBP individual and 2) SKD-induced displacement can affect deeply located anatomical structures. However, further investigation in larger populations is warranted to confirm lumbodorsal tissue displacements and shear strains in the fascia and skeletal muscle of a wide range of subjects. Moreover, it is important to account for confounding factors such as BMI, sex, and age, as these are significantly associated with muscle health in individuals with LBP (Wesselink E. O. et al., 2024).

We have shown that skin displacement causes homogeneous displacement of the fasciae over the deep back muscles. Force transmission from the skin to underlying structures may explain the effectiveness of SKD-induced FTMs. The stiffness of the TLF and surrounding structures may influence tissue displacement during SKD maneuvers. Individuals with LBP may show smaller displacement due to stiffer fascia, interfascial fat, epimuscular fat, and skeletal muscles, but such an effect requires further confirmation. Using state-of-the-art ultrasonography measurement modalities like speckle tracking analysis and shear wave elastography can serve as valuable research tools for analyzing the mechanical properties of fascia and muscles, provided that strict standardized procedures are followed. Speckle tracking analysis of ultrasonography data is a reproducible and accurate method to quantify *in vivo* fascia displacement relative to underlying muscles (Brandl and Schleip, 2025; Van Amstel et al., 2025). The accuracy of anatomical structure displacement measurement is critical, as it forms the primary basis for calculating shear strain between anatomical structures (Langevin et al., 2011). When these pocedures are standardized, ultrasonography provides reliable and reproducible measurements of shear strain and stiffness *in vivo* and *ex vivo* (Creze et al., 2018a; Brandl and Schleip, 2025; Van Amstel et al., 2025).

## Hypothesis 3: The stress induced by SKD during FTMs has the potential to alter the mechanical properties and reduce the nociception of anatomical structures around the spine

Physiotherapists utilize different FTMs to alter mobility, reduce tissue thickness, enhance tissue elasticity, and optimize muscle activity. Upon reviewing FTMs, it becomes evident that in general the skin is displaced and subjected to compression, bending, shear, and tensional forces. As shown and discussed above in Hypothesis 2 on the effects of the SKD maneuver, it is conceivable that SKD induces tensile and shear stress, which is a key component of all FTMs. This transmission of stress from the skin to the underlying structures holds the potential to alter the mechanical properties of the underlying fasciae and skeletal muscles. Therefore, some of the manual FTMs are referred to as myofascial release techniques. However, it is unknown whether indeed myofascial structures are truly released as a result of these techniques.

This chapter outlines the hypothesized impact of various FTMs and how the transmitted force from the skin to deeper structures alters the mechanical properties of fascial tissues. The repeated dynamic and rhythmic SKD-induced FTMs are expected to have distinct effects on tissue stiffness compared to a single SKD maneuver and elastic tape application. Below we discuss several potential mechanisms via which the different FTMs reduce the stiffness of the tissues surrounding the spinal joints.

### The time-dependent viscoelastic mechanical behaviour of fasciae in response to SKD-induced stress

In testing our Hypothesis 2, we revealed *in vivo* that SKD-induced stress causes shear strain of lumbar anatomical structures, which is expected to alter their local stiffness. Stiffness and viscoelasticity are closely linked (Bonfanti et al., 2020), with stiffness referring to the tissue’s resistance to strain under stress and viscoelasticity describing how this resistance to strain changes over time in response to stress, including both elastic (recoil) and viscous (energy-dissipating) properties (Bonfanti et al., 2020).

According to the viscoelastic prediction model for fasciae, SKD-induced stress is expected to change the viscoelastic properties of the fasciae when applied for 60 s (Chaudhry et al., 2007). This means that, with prolonged (≥60 s), consistent local stress applied at or beyond the fascia yield point, the viscosity (η) of the ground substance will decrease (leading to stress relaxation). Simultaneously, the collagen fibers will undergo creep (resulting in a gradual increase in length). This combined effect leads to a decrease in local stiffness (k) and a subsequent increase in local plastic fascia strain over time (Yahia et al., 1993; Schleip et al., 2012a). Indeed, SKD-induced FTMs have been shown to reduce lumbar fascia stiffness by 38%–46%, compared to 10%–21% after electrotherapy, indicating viscoelastic changes like stress relaxation over time (Tamartash et al., 2022c). Additionally, shear wave elastography has demonstrated reductions in left erector spinae muscle stiffness ranging from approximately 12.5 kPa–16.5 kPa following SKD-induced FTMs, whereas control groups showed no such decrease, further supporting the mechanical effects of FTMs on deeper anatomical structures (Devantéry et al., 2023). To gain insight into the changes in viscoelastic behaviour under SKD-induced stress, it is necessary to quantify changes in stiffness of anatomical structures over time (creep and stress-relaxation) and to repeatedly measure stiffness anatomical of structures with rest periods to test hysteresis (loss of elasticity). These are key determinants of viscoelastic changes. Innovative and state-of-the-art ultrasonography imaging techniques, such as elastography or a force transmitter attached to a probe, may potentially be used to test the changes in viscoelastic properties of lumbar anatomical structures (Bartsch et al., 2023), but this is not yet known.

A more commonly used method to study the viscoelastic properties of lumbar anatomical structures is *ex vivo* research on singular isolated lumbar anatomical structures using tensile traction/compression/torque testing machines (Yahia et al., 1993). Studies using these machines in *in-vitro* set-ups also show changes in the viscoelastic behaviour of lumbar skin, superficial fascia, TLF, ESM, and lumbar arthrofascia under tensional loads over time (Yahia et al., 1993; Song et al., 2006; Robertson et al., 2013; Newell and Driscoll, 2024). Hence, both in- and *ex-vivo* studies suggest that SKD-induced stress may affect the viscoelastic properties of lumbar anatomical structures; however, strong evidence *in vivo* is lacking. More extensive in and *ex-vivo* trials are needed to assess the changes in the viscoelastic behaviour of lumbar fasciae and skeletal muscles due to SKD-induced stress.

### SKD-induced stress potentially reduces myofibroblastic and fibroblastic tensile traction forces

Involvement in actively stiffening the fascia through cellular contraction is expected due to the substantial number of myofibroblasts and fibroblasts in the lumbodorsal fasciae (Wilke et al., 2017; Wilke et al., 2018; Schleip et al., 2019a). The tensile traction forces exerted by these cells can potentially be reduced by applying external SKD-induced stress generated during FTMs. Although the contraction force of individual myofibroblasts is low, the summed impact on fascia stiffness might be substantial. Understanding how myofibroblasts exert contractile forces on the fasciae, and how therapeutically applied stress to the skin might reduce or increase cellular traction forces, can offer new perspectives on the role of traction forces in stiffening the anatomical structures surrounding the spine and the mechanisms underlying FTMs.

*In vitro* research has revealed that externally applied stress onto isolated cells reduces the smooth muscle cell, myofibroblastic, and fibroblastic tensile traction forces (a process known as fluidization) (Krishnan et al., 2009; Walker et al., 2020). It is expected that cellular tensile traction forces exerted onto the extracellular matrix instantly decrease in magnitude (Krishnan et al., 2009; Walker et al., 2020). The externally applied tensile and shear stress has been suggested to open the cell’s Ca^2+^ mechanosensitive channels which span the plasma membrane (Sharma et al., 1995; Ng et al., 2005; Godbout et al., 2013) and may cause a Ca^2+^ efflux, thereby reducing cellular traction force (Castella et al., 2010). Other *in vitro* research has shown that after complete fluidization, approximately within 6 min, the (myo)fibroblastic tensile traction force recovers by three different mechanisms: 1) monotonic retraction, 2) reinforced retraction, and 3) monotonic reinforcement (Lee et al., 2012). The full recovery occurs slowly over a period of 25–30 min as a response to mechanical stress (Lee et al., 2012). This recovery may occur through either the Ca^2+^ influx-dependent calmodulin-myosin-light-chain kinase pathway or the integrin-activated Rho kinase pathway, which activates (myo)fibroblasts (Hinz et al., 2001; Tomasek et al., 2002; Levinson et al., 2004).

Fibroblasts and myofibroblasts are responsive to mechanical stimuli, particularly through a feedback control system via the extracellular matrix (Hinz et al., 2019). Potentially, SKD-induced stress could activate mechanosensitive channels in (myo)fibroblasts via the extracellular matrix, causing influx or efflux of Ca^2+^ and altering the cells’ activity state. Both SKD-induced FTMs and elastic tape have the potential to modulate the tensile traction force of (myo)fibroblasts, depending on the local extracellular matrix stiffness. The transmission of stress required to strain the deeper anatomical structures during FTMs and elastic taping depends on the perpendicular and shear stiffness of the superficial fascia. To adequately strain deeper anatomical structures, approximately 100 N of perpendicular force and 22 N of shear force must be generated (Chaudhry et al., 2007). A systematic review concluded that during manual soft tissue mobilizations, a therapist can generate net forces ranging from 232 N to 500 N (Snodgrass et al., 2006). However, elastic tape can generate shear forces of up to approximately 15 N under maximum stretch (Golab et al., 2017). In clinical practice, lower elastic tape stretch levels of 10%–50% are typically used, resulting in substantially less force (van Amstel et al., 2021). Moreover, when deeper structures are stiff, the strain induced by the elastic tape may remain predominantly within the superficial tissues, such as the dermis and superficial fascia, thereby limiting the mechanical effect on deeper anatomical structures. Therefore, it is expected that a therapist applying manual forces can generate sufficient stress to strain deeper anatomical structures, including the embedded cells, whereas elastic tape alone cannot (Chaudhry et al., 2007). Prolonged, consistent stressing of anatomical structures from superficial to deep layers using manual FTMs at the yield point reduces ground substance viscosity (η), leading to stress relaxation, while collagen fibers undergo creep, gradually increasing in length. This leads to a reduction in local stiffness (k), making the fascia and its embedded cells more susceptible to strain. Consequently, applying elastic tape after FTMs might enhance the tape’s ability to induce strain in deeper fasciae and muscles. Future studies should investigate viscoelastic changes in fascia due to SKD-induced FTMs and elastic tape, using advanced ultrasonography techniques (Chen et al., 2021; Soares et al., 2021; Van Amstel et al., 2025), combined with *in vitro* traction force microscopy to quantify myofibroblast tension and elucidate how interactions between extracellular matrix viscoelasticity and myofibroblastic tension contribute to changes in net fascia stiffness (Lee et al., 2012).

### Anti-inflammatory effects of SKD-induced stress

FTMs might alleviate inflammatory responses, increase local blood circulation, and decrease hypoxia. The mechanisms underlying the immunomodulatory effects of FTMs occur at a molecular level by altering the expression of signaling molecules which alter the state of the macrophages (Corey et al., 2012). Inflammation triggers the expression of TGF-β which is a stimulus for collagen production, which is associated with increased cross-link formation, adhesions, and fibrosis (Hillege et al., 2020; Shi et al., 2021). In this section, we will discuss the evidence of immunomodulatory effects of SKD-induced stress generated during FTMs.

In experiments in which mice underwent subcutaneous microsurgical excision of the back muscles, the application of trunk fascial stretching by hanging the mice by their tails (20% strain, 10 min/day, 4 days post-excision) resulted in decreased TGF-β1 levels in these muscles. In addition, with increased trunk fascial stretching (20%–30% strain, 10 min, twice daily, 7 days), the stretched tissue exhibited reduced type-1 procollagen expression levels compared to the untreated group (Bouffard et al., 2008). This implies that SKD-induced stress can reduce inflammation, which leads to a decrease in TGF-β1 levels and collagen type 1 development, which implies that less fibrosis is produced.

Moreover, mice undergoing chemical irritation to induce connective tissue inflammation, showed that SKD-induced stress on connective tissue resulted in a reduction of inflammation (França et al., 2020). In this study, lumbodorsal SKD-induced stress was applied manually using shear loads, tensile loads, and sustained pressure via the skin (intervention duration ≈10 min), which led to significant increases in IL-4 and TGF-β1 levels, while nitric oxide synthase-2 (NOS2) levels were reduced compared to the untreated group (França et al., 2020). SKD-induced stress may cause a decrease in NOS2, which may indicate a reduced production of nitric oxide, which is involved in inflammatory processes, while the increase in IL-4, an anti-inflammatory cytokine, may indicate a reduction in inflammation, which promotes the proliferation of anatomical structures. Also, in rats undergoing chemical-induced inflammatory irritation, it was observed that manually applying pressure, tensile, and shear loads to the lumbar fasciae via the skin, utilizing lumbodorsal instrument-assisted myofascial release techniques on the connective tissue, led to a reduction in inflammation. After myofascial release (5 min, 3 sessions/week, 14 days), there was a decrease in chemokine secretion (RANTES), an increase in neuropeptide-Y, and an increase in IL-10 levels compared to the untreated group (Loghmani et al., 2021). These findings suggest that manually applying pressure, tensile, and shear loads to the lumbar fasciae via the skin may exert an anti-inflammatory effect. This effect is particularly observed through the modulation of TGF-β1, IL-4, IL-10, RANTES, neuropeptide-Y, and NOS2. Improved blood circulation has been suggested to be one of the mechanisms for alleviating connective tissue inflammation due to increased oxygen and nutrient delivery, enhanced removal of metabolic waste products, reduced local fluid stagnation and decreased ischemia (Lokmic et al., 2012; McGarry et al., 2018). Lumbodorsal SKD-induced stress applied to healthy humans significantly increased local blood circulation within lumbar myofascial tissue (31.6% post-treatment, 48.7% at follow-up) compared to the sham group (Brandl et al., 2023a). Improved local blood flow reduces local ischemia which could potentially reduce the risk of hypoxia-induced inflammation, leading to increased oxygen and nutrient delivery. This may help muscles and fasciae heal more quickly from (micro)injuries, resulting in faster recovery from LBP.

These potential anti-inflammatory effects might be crucial in the observed outcomes of SKD-induced stress, yet the immunomodulatory response may vary with the type of SKD-induced stress (shear, tensile, compression). Based on the current literature at least 5 min of SKD-induced stress is necessary to achieve some effect (Loghmani et al., 2021). It is important to note that the timing of immunomodulatory responses seems to be crucial. For example, in some cases, the immune response can be activated to remove waste products and weaken collagen, while in others, it can deactivate the immune response, reduce TGF-β1 levels, and thereby prevent fibrosis development (Bentzinger et al., 2013; Forbes and Rosenthal, 2014).

### FTMs may reduce thoracolumbar fascia stiffness and reduce muscle activity

Ischemia or hypoxia plays a key role in muscle and fascial degeneration, which is characterized by localized changes in fascia and skeletal muscle stiffness, thickness, and linkage density (Lokmic et al., 2012; McGarry et al., 2018). Physiotherapists utilize different FTMs to reduce TLF stiffness, reduce LBP, and improve trunk mobility. However, the effects of SKD-induced FTM and elastic tape application as FTM are expected to differ in their impact on lumbar fasciae and muscles. Therefore, we will discuss the effects of these interventions on fascia stiffness and muscle function.

While one study showed a reduction in TLF thickness after SKD-induced stress in chronic LBP (Tamartash et al., 2022a), another found no thickness change but a decrease in echo intensity, suggesting improved tissue structure without changes in TLF thickness (Yang et al., 2024). In addition to a decrease in TLF thickness, lumbodorsal SKD-induced stress also significantly reduced ESM stiffness (measured by elastography) in individuals with chronic LBP (Devantéry et al., 2023). TLF stiffness has also been reported to change after lumbodorsal SKD-induced stress in healthy individuals (Wong et al., 2017). A similar observation has been reported in individuals with chronic LBP who showed decreased TLF stiffness (using ultrasonography with force transducer), after lumbodorsal SKD-induced stress, lumbodorsal hot pack application, ultrasound therapy, and TENS (Tamartash et al., 2022c). Moreover, lumbodorsal SKD-induced stress and hamstring (dorsal side upper leg) SKD-induced stress application on individuals with chronic LBP revealed that both SKD-induced stress reduced the TLF stiffness independent of the location of SKD-induced stress (ultrasonography with force transducer) (Tamartash et al., 2022b). Collectively, these findings suggest that changes in TLF thickness may be related to TLF stiffness and ESM stiffness. It is important to consider that the effects may vary between locations of treatment and among individuals with or without LBP and that various demographical factors play a role when comparing the study results on TLF stiffness and thickness of the reference studies (Wong et al., 2017; Tamartash et al., 2022c; Tamartash et al., 2022b; Tamartash et al., 2022a; Tamartash et al., 2022d; Devantéry et al., 2023).

Several observational studies (using ultrasonography and MRI) reported that elastic tape applied to the skin can transmit forces in multiple directions and stress and strain the underlying fascia tissues (Pamuk and Yucesoy, 2015; Tu et al., 2016; Wang et al., 2019). Using MRI elastography in healthy individuals, it has been shown that elastic tape application (high elastic tape strain over ESM at heights from sacrum to T12 vertebrae) increased the stiffness of lumbodorsal fasciae and paraspinal muscle underneath the tape (Wang et al., 2019). In healthy individuals, a similar effect of elastic tape applied with medium elastic tape strain over ESM at the height from sacrum to T12 vertebrae has been demonstrated to increase TLF stiffness, measured during trunk flexion (Tu et al., 2016). These results indicate that the application of elastic tape *in situ* tensions the TLF and thereby increases the stiffness of the TLF, potentially enhancing trunk stability during movements such as trunk flexion.

Regarding the effects of lumbodorsal SKD-induced stress on muscle functions, such as muscle activity, voluntary back muscle contractile force, and back muscle stiffness the treatment seems promising to improve lumbar joint mobility. In healthy individuals, lumbodorsal SKD-induced stress caused an increase in LD muscle force exertion determined by a standardized voluntary maximal isometric contraction performed by the lats press-down test (Wong et al., 2017). Furthermore, a decrease in TLF stiffness has been observed during the lats press-down test (Wong et al., 2017). Moreover, the transversus abdominis muscle thickness and its sliding mobility, tested during abdominal contraction, increased significantly after SKD-induced FTM by supposedly targeting the lateral raphe in both healthy individuals and those with LBP (Chen et al., 2016). In individuals with chronic LBP, flexion relaxation following lumbodorsal SKD-induced FTM resulted in reduced ESM activity during trunk flexion compared to a sham-SKD. This might indicate improved muscular control during trunk flexion movements in individuals with chronic LBP (Arguisuelas et al., 2019). However, in contrast to SKD-induced FTM, the application of elastic tape does not affect the ESM activity during trunk flexion in both healthy female individuals and in individuals (male and females) with chronic LBP (Ruggiero et al., 2016; Grzeskowiak et al., 2019). These findings on muscle functions suggest that SKD-induced FTMs may be more effective than elastic taping for improving muscle functions in LBP populations.

In summary, SKD-induced FTMs may cause a decrease in TLF stiffness, affecting the resistance to compression of the lumbodorsal fascia. However, how SKD-induced FTM alters lumbar fascia-muscle shear stiffness remains unknown. Lumbodorsal SKD-induced stress may reduce erector spinae muscle (ESM) activity and increase voluntary LD muscle contractile force. Despite these findings, it is still unknown what the magnitude is of the effects of SKD-induced stress on creep and stress-relaxation (plastic deformation) of the extracellular matrix, as well as on fibroblast and myofibroblast activity, myofascial tensile and shear stress, and the transmission of myofascial force.

Research on the effects of lumbar elastic tape *in situ* on fascia and muscle stiffness suggests that its application increases the stiffness of the TLF and paraspinal muscles. Importantly, there is currently no evidence supporting the notion that elastic tape relaxes muscle activity. However, the instantaneous changes in trunk mobility observed during ongoing direction and location-specific SKD suggest that the effect of elastic tape may also be location and direction-dependent, although this has not been proven in LBP individuals (van Amstel et al., 2022).

In conclusion, both SKD and elastic tape as FTM’s seem to have clinical effects on TLF stiffness and back muscle activity.

### FTMs may enhance the tensile and shear strain between muscles and fasciae required for muscle adaptation during resistance training

In individuals experiencing nonspecific chronic LBP, the observed reduction in TLF shear strain may be a result of or a result in a decrease in mechanotransduction in myofibroblasts, fibroblasts, myofibers, and myoblasts. Considering decreased mechanotransduction on the cellular level, muscle fibers will not be optimally adapted in terms of myofiber size for the required range of motion. FTMs are hypothesized to optimize shear strain between fasciae and muscles and thereby enhance mechanotransduction to cells necessary for the development of typical muscles and fasciae, with mechanical properties required for normal trunk mobility.

Resistance training causes an increase in expression levels of insulin-like growth factor mechano growth factor and its splice variant mechano growth factor, crucial for muscle stem cell activation while myostatin levels decrease (Liu et al., 2008; Jiang Q. et al., 2020) (Figure 4). In addition, in healthy adults resistance training leads to reduced systemic inflammation by lowering cholesterol, triglycerides, low-density lipoprotein, and C-reactive protein (Costa et al., 2019). However, within individuals with LBP, the expression of these growth factors and anti-inflammatory responses during resistance training may be hampered due to the altered mechanotransduction of cells within muscles and fasciae (Purslow, 2010). A possible cause for this impaired responsiveness to resistance exercise-associated mechanical loading is the densification and fibrosis of connective tissue linkages, both epimuscular and intramuscular, resulting in tissue stiffening (Pavan et al., 2014; Wong et al., 2016). Enhanced connective tissue linkage density limits shear strain between the fasciae and back muscles during spinal movements and back muscle contractions (Langevin et al., 2011; Brandl et al., 2022; Brandl et al., 2023b). Increased linkage density between fasciae and muscles reduces shear strain, thereby diminishing mechanotransduction to myoblasts, myofibers, and fibroblasts within these anatomical structures. This may be a key factor in the development of muscle atrophy, fat infiltration into muscle tissue, increased TLF thickness, and the development of fascial and muscle fibrosis (Huijing and Jaspers, 2005; Purslow, 2010; Purslow, 2020).

Achieving an optimal balance between pro- and anti-inflammatory responses is crucial for promoting muscle growth, adaptation (i.e., hypertrophy and addition of sarcomeres in series), and regeneration, as an imbalance can lead to the development of fibrosis within and around the muscles, as well as fat infiltration. FTMs may have the potential to restore the effective force transmission and mechanical interaction between muscles and fasciae, thereby optimizing cellular mechanotransduction required for the expression and secretion of growth factors and cytokines. This restoration of mechanotransduction could enhance the effectiveness of resistance loading-based rehabilitation training in improving tissue regeneration during the rehabilitation of LBP.

### FTMs may enhance sensorimotor control

Physiotherapists use FTMs, either manually or via elastic tape, to restore sensorimotor functions, ultimately enhancing movement patterns. Rat studies have shown that changing muscle and tendon lengths, and tensile and shear tensions within the surrounding fasciae have an impact on reflexes in adjacent skeletal muscles (Maas, 2019). These strains likely activate the Ca^2+^ mechanosensitive channels in the plasma membrane of muscle spindles leading to Ca^2+^ influx or efflux (Boers et al., 2018), resulting in an increase or reduction in muscle contraction, respectively (Bewick and Banks, 2021). Based on these studies, it is hypothesized that in individuals with LBP, SKD as an FTM can instantaneously destress or stress the strained tissue including the sensitized sensors by changing the relative position between tissues (i.e., muscles and fasciae) (Figure 6C). This modulation of sensorimotor control is hypothesized to acutely influence muscle activity and as such joint range of motion providing a potential explanation for the observed instantaneous effects of SKD on joint mobility in healthy individuals (van Amstel et al., 2022).

In addition to the instantaneous effects of SKD-induced stress on changes in relative position between the TLF, back muscles, and spine, influencing muscle activity and trunk range of motion, SKD-induced stress likely also affects tactile sensation-induced changes. Touching the skin has an additional advantage compared to the sensory input elicited by pleasant touch, which can be an important factor contributing to the tactile effect of SKD (Olausson et al., 2016). Tactile C fibers in the dermis, subcutis, and other connective tissues have been shown to be related to the sensation of pleasant touch (Pawling et al., 2017). Moreover, deep pressure at the lower leg (70 mmHg) was not associated with the activity of the primary motor cortex in healthy adult individuals, but rather with that of the insula (Case et al., 2021). Hence the epi-and intramuscular interoceptors are presumably responsible for the pleasant pressure experienced (Case et al., 2021) which may explain the psychological effect of touch during SKD on pain sensation. Nevertheless, the instantaneous mechanical effects of SKD-induced stress are expected to be associated with changes in neuromuscular reflexes rather than with psychological effects.

### FTMs may reduce pain intensity and increase trunk mobility

Effects of SKD-induced FTMs on pain intensity have been investigated in a meta-analysis (Wu et al., 2021). Overall, SKD-induced FTMs showed a significant reduction in pain intensity compared to control groups. Subgroup analyses indicated that SKD-induced FTMs were notably more effective than sham interventions. These results suggest that SKD-induced FTMs alone can effectively reduce pain intensity in individuals experiencing LBP (Wu et al., 2021). An SKD-induced FTM intervention study demonstrated that reductions in pain intensity were associated with decreased TLF stiffness (Tamartash et al., 2022c). This suggests that reducing TLF stiffness might modulate nociceptors within the TLF (Taguchi et al., 2009; Tesarz et al., 2011; Mense, 2019). In contrast to SKD-induced FTMs, lumbar elastic tape does not result in pain reduction, as confirmed by a meta-analysis (Li et al., 2019).

Trunk mobility was also evaluated in another meta-analysis of LBP. The combined findings revealed that SKD-induced FTMs did not lead to a significant improvement in trunk mobility compared to sham FMTs (Wu et al., 2021). Similar results were observed for lumbar elastic tape application, which did not significantly improve trunk mobility, when taking into account? The measurement error of the instrument used to test changes in trunk mobility (van Amstel et al., 2021).

In summary, SKD-induced FTMs for reducing pain intensity in LBP individuals, show significant pain reductions compared to controls and sham interventions. This suggests that SKD-induced FTMs effectively alleviate LBP. Reductions in pain intensity were related to decreased TLF stiffness, potentially deactivating nociceptors. In contrast, no significant improvement in trunk mobility with SKD-induced FTMs or lumbar elastic tape application occurred indicating limited effectiveness in enhancing trunk mobility in LBP.

### Conclusion and perspectives regarding the underlying mechanisms of FTMs in modulating fascia-muscle stiffness, muscle reflexes, pain intensity, and trunk mobility

Although the evidence presented in the preceding sections is based on studies conducted *in vivo*, *ex vivo*, and *in vitro*, involving both animals and humans, it is important to note that the proposed hypotheses need additional studies for exploration and testing. A comprehensive understanding of the relationships between changes in fasciae stiffness, changes in muscle function, and joint mobility in humans is essential not only to understand the rationale to apply but also to optimize the effect of FTMs. This also includes a more comprehensive understanding of the molecular responses at the cellular level, such as those in myofibroblasts, fibro-adipogenic progenitors, myoblasts, macrophages, and fibroblasts, to SKD-induced mechanical stress. In addition, the viscoelastic behaviors of these tissues (muscles and fasciae) were investigated in *ex vivo* animal and human specimens when fully dissected free from their surroundings (Yahia et al., 1993; Schleip et al., 2012a; Newell and Driscoll, 2024). Note that the dissection of fascia or muscle impacts its mechanical interaction with adjacent structures and as such it properties. The challenge lies in integrating outcomes of *in vivo* and *ex vivo* experimental tests on distinct anatomical structures such as fascia, muscle and skin into coherent mathematical models that capture their individual and interactive biomechanical behaviors. This approach aims to derive realistic estimates of various factors influencing mobility limitations while considering variables such as age, sex, weight, length, hydration levels, and temperature. Examining the molecular mechanisms underlying SKD-induced stress responses holds promise for broader implications and potential therapeutic applications of FTMs. The most likely mechanism of FTMs is that SKD transmits force to the underlying anatomical structures. These forces put the anatomical structures under tensional and shear stress, which can cause two responses: a connective tissue response and a cellular response. Connective tissue will exhibit stress-relaxation and creep, and the contractile activity of skeletal muscle cells and myofibroblasts will decrease, both responses leading to a reduction in overall stiffness (Figure 1). These effects are time-dependent in which acute, subacute, and long-term effects can be distinguished. Further research is required to assess the magnitude of these contributions in general and individually in each LBP patient.

Generally, variable effects have been reported regarding SKD-induced FTMs and elastic tape application. Overall findings suggest that SKD-induced FTMs may be more effective than elastic taping for reducing TLF stiffness, improving muscle activity, increasing trunk mobility, and reducing pain in LBP individuals. The literature reports that the effect of both SKD-induced FTMs as elastic tape is location and direction-dependent (Pawlukiewicz et al., 2022; Van Amstel et al., 2023). These claims are supported by the observation that shear strains resulting from SKD maneuvers are depth and direction-specific, as demonstrated in Hypothesis 2. Further research is necessary to establish a definitive understanding of the clinical implications of different FTMs as interventions.

## Clinical implications for diagnostics and treatment

Fascia tissue manipulations have become very popular in the treatment of LBP, especially among physiotherapists.

The literature demonstrates that SKD-induced FTMs effectively reduce TLF stiffness, improve trunk mobility, and decrease pain intensity in individuals with LBP. In this review, we have proposed various underlying working mechanisms for SKD-induced FTMs. The effects depend on the amount, of stress and the duration of the applied SKD. Research is needed to understand precisely which direction, dosage of stress, and duration are needed to optimize SKD-induced FTMs.

Regarding elastic tape, there is a lack of studies regarding its effect on lumbar fasciae and muscle stiffness. Specifically, there is limited research on how lumbar fasciae and muscle stiffness change when the elastic tape has been *in situ* for a certain period. Moreover, the literature cannot support the effect on pain reduction and increase in spinal mobility. The literature debates that the current effects of lumbar elastic tape application methods are not effective. In contrast, SKD has been shown to influence spinal mobility immediately, depending on the SKD location and direction. This raises the hypothesis that applying elastic tape in a similar direction to effective SKD might yield comparable outcomes in terms of pain and mobility. Hence, in clinical practice, it seems advisable to test which locations and directions of the FTMs will be most effective (e.g., the DAMT-Test). However, further evidence is needed to support its psychometric qualities.

In line with the perspectives outlined in this review, we emphasize the importance of a standardized and well-documented approach in clinical practice. This includes precise documentation of the FTMs applied in RCTs and other clinical trials. We also support the collection of detailed patient information to better understand the effect of different treatment modalities with FTMs as an add-on, in relation to variability in patient characteristics and treatment outcomes. This standardized approach helps to build a solid foundation for developing individualized treatment strategies for LBP.

## Limitation

This narrative review ensures transparency by using predefined search terms per hypothesis. While we aimed for a comprehensive and balanced discussion, we acknowledge that not all relevant research has been covered. A key strength is the inclusion of both supportive and critical studies, avoiding selective reporting. In addition, we did not include studies targeting the arthrofascia, including joint (impulse) mobilizations, which are considered FTMs according to the definition. Future research should update the search strategy to reflect the latest developments regarding these manual interventions.

## General conclusion

This review provides an overview of the literature and explores the mechanisms underlying the positive effects of FTMs on fascia and muscle function in alleviating LBP. It discusses manual FTMs, such as soft tissue mobilizations, as well as the non-manual FTM of elastic tape, with conclusions drawn from *in vitro*, *in vivo*, and *ex vivo* studies.

The following findings support the proposed hypotheses: 1) The skin (epi)dermis is closely connected to the superficial fascia, deep fascia, back muscles, and lumbar arthrofascia via the CIFT. Increased lumbar fascial thickness and linkage density between anatomical structures contribute to fascial stiffness, limited thoracolumbar fascia displacement, altered myofascial force transmission, and reduced shear stress in the fascia and muscles. These changes may affect muscle activity during joint motion in individuals with LBP, leading to variations in muscle activation depending on the specific muscle involved. 2) Our dissection and ultrasonography findings show that SKD maneuvers induce tensile and shear strains in deeper tissues, displacing the thoracolumbar fascia, latissimus dorsi, and serratus posterior inferior, and causing them to slide over the ESM. This is accompanied by increased tension on the arthrofascia due to elevated strain on the CIFT. This can lead to positional changes among the back muscles and potentially high shear stress between them. 3) SKD-induced FTMs likely alter the mechanical properties of connective tissues, triggering cellular responses that reduce fascia and muscle stiffness, promote fascia sliding mobility, and improve capacity for straining under SKD-induced stress. Reducing fascia stiffness might optimize tensile and shear stress between muscles and fasciae, aiding mechanotransduction in cells and facilitating adaptation during resistance training. Moreover, improving thoracolumbar fascia stiffness and sliding mobility may change the relative positions of the fascia, lumbar spine, and muscles, influencing sensory cell activity, reducing nociceptive cell activity, and normalizing muscle reflexes in individuals with LBP.

This review alongside experimental results provides a theoretical framework that could explain the working mechanism of FTMs. Extensive research is required to further investigate these mechanisms as there is currently a significant knowledge gap. Further research is needed to bridge the knowledge gap and optimize FTMs for improving muscle function, spinal mobility, and pain relief, all of which are crucial for the individual patient.

## Data Availability

The raw data supporting the conclusions of this article will be made available by the authors, without undue reservation.

## Abbreviations

FTMs, Fascia Tissue Manipulations; Manual FTMs, skin displacement, soft tissue mobilization, myofascial release, rolfing, joint (impulse)mobilizations, etc.; Non manual FTMs, elastic tape application, foam rolling, vibration therapy, etc.; SKD, Skin Displacement; CIFT, Central Interfascial Triangle; TLF, Thoracolumbar Fascia; ESM, Erector Spinae Muscles; ESA, Erector Spinae Aponeurosis; SPI, Serratus Posterior Inferior; DAMT-Test, Dynamic ArthroMyoFascial Translation Test; LBP, Low Back Pain.
