# Supplementary material for: A review and empirical findings of fasciae and muscle interactions in low back pain
Source: Front Physiol. 2025 Sep 10;16:1604459. doi: 10.3389/fphys.2025.1604459 (PMC12457458; doi:10.3389/fphys.2025.1604459)
Supplement: Supplementary file 1 [file Supplementaryfile1.docx]

# **Supplement S1: Research questions and Search strings**

Each hypothesis will be addressed by discussing the current scientific evidence. These hypotheses are approached through a narrative review, where we use search strings to assess whether the identified studies provide relevant information considering the research questions. We formulated the research questions and corresponding search strings (S1), and searched PubMed and Google Scholar for studies published between 2000 and 2025. The first 100 results were screened for relevance to the research questions. Additionally, references from identified papers were also reviewed for useful information (1^st^ and last authors). Each research question concludes with a summary of findings, highlighting evidence gaps and suggestions for future research.

Below, we outline the **research questions** and corresponding **search strategies**:

1. **What are the anatomical characteristics and linkages of the lumbodorsal region?**
   - Search string: (((((Skin[MeSH Terms]) OR (subcutaneous tissue[MeSH Terms])) OR (superficial fascia[MeSH Terms])) ) AND (anatomy and histology[MeSH Terms])) OR (biomechanics[MeSH Terms])
   - Search string: ((thoracolumbar fascia) OR (anatomy and histology[MeSH Terms])) OR (biomechanics[MeSH Terms])
   - Search string: ((myofascia fascia) OR (anatomy and histology[MeSH Terms])) OR (biomechanics[MeSH Terms])
   - Search string: ((spine[MeSH] AND anatomy[MeSH Terms] OR histology [MeSH] OR biomechanics[MeSH Terms])
   - Additional terms: "cadaver," "magnetic resonance imaging"
2. **How does the morphology of lumbodorsal fascial tissue change, specifically in terms of thickness?**
   - Search string: (((lumbar region[MeSH Terms]) AND (fascia[MeSH Terms])) ) AND (morphology)
   - Additional terms: "thickness,"
3. **What is the impact of lumbar epimuscular tissue thickness on lumbodorsal fascial mechanics in patients with low back pain?**
   - Search string: (((lumbar region[MeSH Terms]) AND (fascia[MeSH Terms])) ) AND (morphology)
   - Additional terms: "shear strain," "stiffness," "elastic modulus"
4. **Can the skin act as an epimuscular myofascial force transmission pathway, and how does it influence underlying tissues?**
   - Search string: ((((Skin)) OR (Superficial fascia)) OR (epimuscular) AND (Force))
   - Additional terms: "transmission," "deformation," "stress-strain"
5. **What are the effects of fascial tissue manipulation on fascial and muscle mechanics?**
   - Search string: (((((low back pain[MeSH Terms]) ) AND (therapy)) AND (stiffness)) OR (elastography)) OR (elastic modulus)
   - Additional terms: "myofascial release," "kinesiotape," "kinesio tape"
6. **What is the potential impact of SKD on the tensile traction force of fibroblasts, myoblasts, and myofibroblasts within the fasciae and skeletal muscles?**
   - Search string: ((((((myofibroblasts[MeSH Terms]) OR (fibroblasts[MeSH Terms])) OR (myoblasts, skeletal[MeSH Terms])) AND (contraction)) OR (stiffness)) AND (fascia[MeSH Terms])) OR (fiber, skeletal muscle[MeSH Terms])
7. **How do fibrous matrices respond to stress in terms of creep and stress relaxation?**
   - Search string: ((fascia[MeSH Terms]) AND (biomechanics[MeSH Terms])) OR (viscoelastic)
   - Additional terms: "kinematics", "spine"
8. **How does the sensorimotor reflex system respond to relative position changes in the fascia?**
   - Search string: (((mechanoreceptor cells[MeSH Terms]) AND (innervation)) AND (fascia[MeSH Terms])) OR (fibers, skeletal muscle[MeSH Terms]) Additional terms: "painful," "pleasant," "tactile," "touch," "pressure"
