# Supplementary material for: A review and empirical findings of fasciae and muscle interactions in low back pain
Source: Front Physiol. 2025 Sep 10;16:1604459. doi: 10.3389/fphys.2025.1604459 (PMC12457458; doi:10.3389/fphys.2025.1604459)
Supplement: Supplementary file 2 [file Supplementaryfile2.docx]

# **S2. Testing Hypotheses on Lumbodorsal Linkages and SKD-Induced Stress Transmission: Cadaver and In Vivo Analyses**

Robbert van Amstel^a,b,e*^, Guido Weide^a^, Eddo Wesselink^b^, Karl Noten^e^, Karl Jacobs^c,d^, Annelies Pool-Goudzwaard^b,f^, Richard Jaspers^a,b^

## ***Testing hypothesis 1: Dense lumbodorsal linkages observed from the skin to the spine in a cadaver and increased in vivo fascial thickness in two individuals***

To investigate potential chronic LBP-related changes and adaptations of lumbodorsal connective tissues and the linkages between them, we assessed the different fasciae in the lower back regions in vivo and in a cadaver specimen (ex vivo) by using three different observation techniques: 1) segmentation of 3D images of an anatomical model (Visible Human Male (NIH, 2013)), 2) visualization of lumbodorsal fasciae and muscles in a fresh-frozen cadaver, and 3) 2D ultrasonography analysis. The 2D ultrasonography images were obtained from a non-embalmed male freshly frozen cadaver and from 2 male subjects, one with (age 39, 178, 78 kg, BMI 24.5) and one without LBP (age 22, 189, 83 kg, BMI 23.2) using a 12-2 MHz linear array transducer (Arietta Prologue; Hitachi Ltd., Tokyo, Japan). The fresh-frozen cadaver was stored at −20°C and was thawed at room temperature 24 hours before the investigation. This study was approved by the Scientific and Ethical Review Board (METC) of the VU University Medical Center Amsterdam, Vrije Universiteit Amsterdam (CTB- 2017.098 (A2017.457)).

The 3D anatomical model segmentation and the dissection of the fresh-frozen cadaver revealed a strong connection between the skin (epidermis, dermis), superficial fascia **(Figure 7)**, and the superficial lamina of the posterior layer of the TLF, spinous processes, and supraspinal ligaments **(Figure 8)**. At the location of the lumbar vertebra (T12-L5) within the hypodermis, the lumbar superficial fascia forms a triangle-shaped septum including a dense collagenous microvacuolar network **(Figure 8, 9)**. The triangular-shaped structure is referred to as the lumbodorsal central interfascial triangle (CIFT). The apex of the CIFT points anteriorly toward the spinal processes/supraspinal ligaments where it is attached to these tissues, whereas the basis of the CIFT points towards the dermis (**Figure 8, 9)**. The CIFT exists approximately between the distal end of the longissimus muscles where the CIFT merges with both the superficial-and profundal (deep) lamina of the posterior layer of the TLF. The superficial lamina of the posterior layer of the TLF is closely connected to the aponeurosis and epimysium of the latissimus dorsi (LD) and the serratus posterior inferior muscles (SPI). This connection merges with the superficial and deep lamina of the posterior layer of the TLF **(Figure 8A)**. The CIFT should not be mistaken for the lumbar interfascial triangle also known as the lateral raphe **(Figure 8A)** described in the literature (BOGDUK and MACINTOSH, 1984). The lateral raphe can be found within the TLF along the lateral border of the paraspinal muscles from the 12^th^ costal arch to the iliac crest. The lateral raphe is a triangular-shaped structure that surrounds the paraspinal muscles and merges with the aponeuroses of the transversus abdominis and the internal oblique muscles **(Figure 8A)** (Schuenke et al., 2012).

Between the superficial-and deep lamina of the posterior layer of the TLF, an interfascial fatty connective tissue layer was observed, which separates both laminae from each other **(Figure 8B, C)**. The interfascial fatty connective tissue present between the posterior TLF laminae is thicker at the medial aspect of the spinal process and thinner more laterally **(Figure 8B)**. Moreover, an epimuscular fatty connective tissue layer was found between the deep lamina of the TLF and the ESA **(Figure 8C, D)**. During dissection in the interfascial and epimuscular fatty connective tissue, dense fibrous tissues were observed linking the laminae of the TLF and the ESA with the TLF. Moreover, the epimuscular fatty connective tissue was strongly linked to the TLF and loosely attached to the ESA.

Using ultrasonography, the thickness of the lumbodorsal hypodermis (including the superficial fascia) and TLF by LBP and healthy cases were measured by calculating the sagittal cross-sectional area **(Figure 10)** with the ImageJ (Rasband, 2011). The mean cross-sectional area of the lumbodorsal tissues in the ultrasonography showed that in the healthy case, the CSA of the hypodermis including the superficial fascia was 258 mm^2^ and in the LBP case was 243 mm^2^. The CSA of the TLF in the healthy case was 90 mm^2^, and in the LBP case, it was 122 mm^2^. The thickness measurement was based on three repeated measurements performed by the 1^st^ author which were found reliable (ICC= .96). The larger CSA of the TLF in the LBP case potentially reflects the thickening of both the TLF laminae as well as the interfascial, and the epimuscular fatty connective tissues, previously mentioned as highly attached to the TLF.

The cadaver dissection, anatomical model segmentation, and ultrasonography analysis of the lumbodorsal tissues confirm dense lumbodorsal linkages between the skin, TLF, superficial back muscles, and lumbar spine. In contrast, loose lumbodorsal linkages between the TLF and ESM are confirmed due to a loose epimuscular connective tissue layer. The observed thickened TLF in patients with LBP suggests that the TLF and the loose epimuscular connective tissue layer should be the subject of further study. Detailed assessment of the linkages between paraspinal muscles and TLF and related thickness is essential for understanding LBP.

## ***Testing hypothesis 2: Shear strain analysis by ultrasonography reveals SKD-induced transmission of stress to underlying structures, providing proof of the SKD principle***

To demonstrate that SKD-induced stress causes transmission of stress from the skin to the underlying structures, SKD maneuvers were performed on the above-described individuals and one cadaver specimen (see for details testing hypothesis 1). Four SKD maneuvers were performed at location L3 by a physiotherapist with 11 years of experience in using FTMs and performing the DAMT-Test (Noten, 2021). The SKD maneuvers consisted of a medio-lateral directed SKD to the Right (R) or Left (L) direction with respect to the spine at the location L3 (RL3, LL3) followed by a vertically directed SKD maneuver performed in an upward or downward direction above the right ESM. The SKD maneuvers were performed in neutrally and anteriorly flexed positions. The two individuals were asked to inhale (using their own pace) and exhale in 4 seconds, followed by one of the SKD maneuvers. The SKD maneuver was performed by placing the thumbs bilaterally of the ESM at the height of L3. For example, in SKD RL3, the left thumb generated a pushing force, while the right thumb generated a pulling force. Be aware that the skin is also compressed to get a hold of it. Simultaneously, the skin is displaced mediolaterally to the right in 4 seconds and held at the end of motion for 4 seconds. The SKD maneuver intensity was adjusted to the skin slack.

Effects of SKD-induced stress on the underlying structures were assessed by ultrasonography measurements of the right side. Ultrasonography images were recorded above the LD and ESM in the sagittal and transverse planes. Within the ultrasonography images, the following anatomical structures were localized: lumbodorsal dermis, superficial fascia, TLF, m. latissimus dorsi (LD), m. serratus posterior inferior (SPI), ESA, and ESM. Real-time ultrasonography images were validated on the freshly frozen cadaver through dissection **(Figures 7, 8, 11)**. Agreement on the identification of anatomical structures was high between and within the two operators who performed the standardized ultrasonography protocol in finding the anatomical structures (*k* =.96).

The recorded ultrasonography videos were post-analyzed. Displacements of pre-defined anatomical structures from superficial to deeper layers were selected **(Tables 1 and 2)**. At the regions of interest, speckles were tracked using Kinovea (Puig-Diví et al., 2019), in a standardized manner. The sliding mobility was determined, using a custom-made MATLAB script (The MathWorks, Natick, MA, USA). The absolute tissue displacement of each structure was quantified using the following equation:

$${Tissue}_{displacement}\int_{0}^{10} \Delta{distance}_{({Tissue}_{Position 2}-{Tissue}_{Position 1})} \left( t \right)dt$$

Subsequently, the shear strain ratio between the dermis and each underlying anatomical structure was calculated using the formula reported in the literature (Langevin et al., 2011):

$Shear strain ratio= \frac{|dermis-anatomical structure|\times100}{{Depth}_{centroid ROI}}$

The tracked speckles indicated that SKD-induced stress caused displacements of the underlying pre-defined anatomical structures. In both planes, the magnitude of shear strain ratios between the (epi)dermis, superficial fascia, and the posterior layer of the TLF was small, suggesting equal displacement of these layers in the same direction. In contrast, higher shear strain ratios were observed between the (epi)dermis and various back muscles, indicating greater relative displacement and potential shear stress between the TLF and the back muscles.

The LD and SPI were also displaced in the direction of the SKD-induced stress. The displacement followed a hierarchical pattern, with the greatest displacement occurring superficially and gradually decreasing in depth **(Figure 11)**. The superficial TLF lamina displaced mediolaterally over the deep TLF lamina by mediolateral SKD-induced stress but was almost not displaced in caudal-cranially direction. The deep TLF lamina did slide over the ESA in mediolateral and caudal-cranial directions. In general, the displacements of all pre-defined anatomical structures in the transverse plane were substantially greater than in the sagittal plane in which the displacements and shear strain ratios of these anatomical structures were reduced substantially in trunk flexion position in both planes **(Tables 1 and 2)**. Moreover, the displacement of the anatomical structures due to the SKD maneuver was greatest in the healthy individual in comparison to the LBP individual and cadaver specimen. In addition, the SKD on the dissected cadaver specimen revealed that the stress applied through SKD caused the superficial fascia and TLF to slide over the ESA due to the mobile epimuscular loose fatty connective tissue. This is accompanied by increased tension on the spinal arthrofascia due to elevated strain on the CIFT. This observation further supports the ultrasonography findings, confirming the transmission of force from the skin to the underlying anatomical structures, and facilitating the displacement of the TLF, LD, and SPI over the ESM. However, we do have to consider that even in fresh cadavers the biomechanical properties of different tissues can be different from in-vitro.

These ultrasonography observations show changes in relative positions among the TLF, LD, SPI, ESM, and lumbar spine with respect to each other indicating the force transmission between the anatomical structures and representing a key element in the mechanisms underlying the effectiveness of SKD-induced FTMs. Based on these findings, we anticipate that forces required for TLF strain and/or displacement during SKD-induced FTMs depend on the stiffness of the TLF, as well as the stiffness of the structures lying superior and inferior to it, along with their linkage density. As in individuals with nonspecific LBP these structures may have stiffened, it is expected that these individuals undergoing SKD-induced stress would experience less TLF, LD, and SPI displacement relative to the ESM and lumbar spine than healthy individuals. However, whether this is the case needs to be confirmed.

Here we provide evidence that transmission of stress occurs from the skin to the underlying anatomical structures in response to SKD-induced stress. Although this evidence is limited by the small sample size, to the best of our knowledge, this is the first observation demonstrating that SKD maneuver-induced displacement affects anatomical structures deeply located beneath the skin. Our results show that the shear strain between the skin and underlying anatomical structures was smaller in the LBP subject than in the healthy subject. Since this was based on two cases this may not be representative of LBP individuals in general. Nevertheless, it supports the notion that in subjects with LBP, the stiffening of structures below the site of skin displacement due to fascial shear strain during SKD maneuvers will be reduced. Future studies should investigate tissue displacements and shear strains of deeper anatomical tissues due to SKD-induced stress in a large study population (healthy and LBP). The use of real-time ultrasonography assessment and post-analyzing of ultrasonography videos in analyzing the mechanical behavior of the lumbodorsal muscles and fasciae in vivo seems promising.

# **References**

BOGDUK, N., and MACINTOSH, J.E. (1984). The applied anatomy of the thoracolumbar fascia. *Spine* 9(2)**,** 164-170.

Langevin, H.M., Fox, J.R., Koptiuch, C., Badger, G.J., Greenan-Naumann, A.C., Bouffard, N.A., et al. (2011). Reduced thoracolumbar fascia shear strain in human chronic low back pain. *BMC musculoskeletal disorders* 12(1)**,** 203.

NIH (2013). "National Institute of Health. The visible human project".).

Noten, K. 2021. The Dynamic ArthroMyofascial Translation® Test (DAMT®Test): 2012. (4xT®Method the ArthroMyofascial Therapy: Chapter 2). Available: <https://doi.org/10.17605/OSF.IO/D85K3> [Accessed December 3].

Puig-Diví, A., Escalona-Marfil, C., Padullés-Riu, J.M., Busquets, A., Padullés-Chando, X., and Marcos-Ruiz, D. (2019). Validity and reliability of the Kinovea program in obtaining angles and distances using coordinates in 4 perspectives. *PloS one* 14(6)**,** e0216448.

Rasband, W.S. (2011). Imagej, us national institutes of health, bethesda, maryland, usa. [*http://imagej*](http://imagej)*. nih. gov/ij/*.

Schuenke, M., Vleeming, A., Van Hoof, T., and Willard, F. (2012). A description of the lumbar interfascial triangle and its relation with the lateral raphe: anatomical constituents of load transfer through the lateral margin of the thoracolumbar fascia. *Journal of anatomy* 221(6)**,** 568-576.
